# Investigation of DNA sequence recognition by a streptomycete MarR family transcriptional regulator through surface plasmon resonance and X-ray crystallography

**DOI:** 10.1093/nar/gkt523

**Published:** 2013-06-07

**Authors:** Clare E. M. Stevenson, Aoun Assaad, Govind Chandra, Tung B. K. Le, Sandra J. Greive, Mervyn J. Bibb, David M. Lawson

**Affiliations:** ^1^Department of Biological Chemistry, John Innes Centre, Norwich Research Park, Norwich NR4 7UH, UK and ^2^Department of Molecular Microbiology, John Innes Centre, Norwich Research Park, Norwich NR4 7UH, UK

## Abstract

Consistent with their complex lifestyles and rich secondary metabolite profiles, the genomes of streptomycetes encode a plethora of transcription factors, the vast majority of which are uncharacterized. Herein, we use Surface Plasmon Resonance (SPR) to identify and delineate putative operator sites for SCO3205, a MarR family transcriptional regulator from *Streptomyces coelicolor* that is well represented in sequenced actinomycete genomes. In particular, we use a novel SPR footprinting approach that exploits indirect ligand capture to vastly extend the lifetime of a standard streptavidin SPR chip. We define two operator sites upstream of *sco3205* and a pseudopalindromic consensus sequence derived from these enables further potential operator sites to be identified in the *S. coelicolor* genome. We evaluate each of these through SPR and test the importance of the conserved bases within the consensus sequence. Informed by these results, we determine the crystal structure of a SCO3205-DNA complex at 2.8 Å resolution, enabling molecular level rationalization of the SPR data. Taken together, our observations support a DNA recognition mechanism involving both direct and indirect sequence readout.

## INTRODUCTION

MarR family transcriptional regulators (MFRs) are widespread across bacteria and archaea, where they regulate various functions, including the response to oxidative stress (1–3), pathogenesis (4,5) and antibiotic resistance (6,7), and are thus of significant clinical interest. Moreover, the abundance of MarR proteins in the streptomycetes, which are the source of many therapeutic compounds, raises the prospect of exploiting them to influence the natural product profiles of these soil bacteria. MFRs are one-component response regulators, having input and output domains fused in a single protein chain. They function as homodimers and regulate gene expression by binding to pseudopalindromic DNA sequences through winged-helix DNA-binding motifs. Most MFRs are repressors, and their abilities to bind DNA are abrogated by the binding of small molecule ligands or by sensing reactive oxygen species via reactive cysteine residues (8). Herein, we present a generic and integrated study that combines bioinformatics, surface plasmon resonance (SPR) and X-ray crystallography to predict, verify and footprint operator sites for a streptomycete MarR family transcription factor and subsequently to biophysically characterize these protein–DNA interactions.

Many bacterial transcription factors negatively autoregulate (9). Thus, their upstream intergenic regions are suitable places to search for their operator sites. Within these regions, there will be strong evolutionary pressure to conserve sequences that are important for regulation; elsewhere, the sequences will tend to be more variable. The availability of in excess of a hundred actinomycete genome sequences allows us to use bioinformatics to make robust predictions about the operator sites for widely represented transcription factors through a comparison of their upstream sequences with those of their orthologs. Using this approach, we identified two potential operator sites upstream of *sco3205*, which encodes a putative MFR, one of 42 in the *Streptomyces coelicolor* genome.

SPR is well-suited to the study of protein–DNA interactions and offers several advantages over more traditional *in vitro* methods that only provide affinity data under equilibrium conditions, such as electrophoretic mobility shift assays or nitrocellulose filter binding assays, because it can provide real-time monitoring of binding and dissociation events, enabling rapid measurement of stoichiometric, kinetic, affinity and thermodynamic parameters (10–12). Furthermore, the technique is sensitive and highly automatable. The most convenient approach to study protein–DNA interactions by SPR is to tether the DNA (the ‘ligand’) via a biotin tag to a streptavidin (SA)-coated chip and then to flow the protein (the ‘analyte’) over the top to monitor the interaction. One drawback of this method is that once the biotinylated DNA has been captured on the chip surface, the interaction is so tight that it cannot be removed without damaging the chip. Moreover, if the protein–DNA interaction is strong, it may not be possible to remove the protein without damage to the bound DNA or to the chip surface. As the sensor chips are costly, we used an indirect capture strategy, based on previous work (13–15), that enables the chip to be reused. The method uses a biotinylated single-stranded (ss) DNA linker, which is permanently bound to a standard SA chip. Subsequently, a double-stranded (ds) DNA oligomer containing the sequence of interest, and bearing an overhang that is complementary to the linker, is captured on the chip through hybridization. At the end of the experiment, the captured oligonucleotide is stripped from the linker by denaturation to regenerate the chip. Surprisingly, this approach has not seen widespread application, possibly because it was reported to be less stable than the conventional direct capture procedure (15). By contrast, in our hands, we show that this *R*e-usable *D*NA *Ca*pture *T*echnique (ReDCaT) is robust and enables the chip surface to be repeatedly and reliably regenerated, whilst yielding data of comparable quality to those obtained using standard direct ligand capture. Moreover, we have developed generic SPR methods based on ReDCaT, which we use to validate and precisely footprint the two SCO3205 operator sites that we predicted by bioinformatics. Comparison of these sites reveals a consensus sequence of TTnAAnnnTCAA that enables us to identify three further potential operators in the *S. coelicolor* genome. Using ReDCaT, we assess the validity of these additional sites and test the importance of the conserved bases within the consensus motif. In performing these experiments, we use a single SA chip in excess of 450 times, clearly demonstrating the cost-effectiveness of ReDCaT.

Guided by the operator sequences, we determined through SPR footprinting, we go on to crystallize and solve the X-ray structure of a SCO3205-DNA complex at 2.8 Å resolution. This provides molecular-level understanding to the bioinformatics and SPR results. In particular, it endorses the SPR footprinting and enables us to rationalize the roles of specific bases with the consensus motif. Moreover, the structure reveals an ordered C-terminal tail for SCO3205, not seen in any MFR-DNA complex thus far, that plays an important function in mediating protein–DNA interactions.

## MATERIALS AND METHODS

### Expression and purification of SCO3205 protein

The *sco3205* coding sequence (http://www.ncbi.nlm.nih.gov/gene/1098639) was chemically synthesized and codon optimized (GeneArt) for expression in *Escherichia coli*. The 163-residue native sequence was synthesized with the addition of a Histidine tag and a tobacco etch virus (Tev) protease cleavage site to the N-terminus (total deduced molecular mass of 20523 Da; the full nucleotide sequence of the synthetic gene is shown in Supplementary Figure S1). The construct was then subcloned into pET21a to give the expression vector pET21a-*sco3205*. This was transformed into *E. coli* strain BL21 (DE3), and a 10 ml of overnight culture of the cells was used to inoculate 1 l of Luria-Bertani media containing 100 µg ml^−^^1^ ampicillin. The cells were grown at 37°C to an optical density at 600 nM of ∼0.4. The temperature was reduced to 25°C, and expression was induced by the addition of isopropyl β-D-thiogalactopyranoside to a final concentration of 1 mM. After shaking overnight at 200 rev min^−^^1^, the cells were harvested by centrifugation. The cell pellet was resuspended in buffer A [50 mM phosphate (pH 8), 300 mM NaCl, 40 mM imidazole] containing a Complete EDTA-free protease inhibitor cocktail tablet (Roche) and lysed by two passes through a French press. The cell debris was removed by centrifugation and the supernatant applied to a 1 ml nickel-charged HisTrap chelating column attached to an Äkta FPLC (GE Healthcare). The column was then washed with buffer A to remove any unbound proteins, and the bound protein was eluted with a linear gradient to 750 mM imidazole in buffer A. The fractions containing SCO3205 protein (confirmed by SDS–PAGE) were pooled, EDTA was added to a final concentration of 10 mM and the sample was concentrated to ∼5 mg ml^−^^1^ using an Amicon Ultra-4 10 kDa cutoff concentrator (Millipore). This was filtered through a 0.2 µm filter (Sartorius) before loading on to a Superdex 75 Hiload gel filtration column attached to an Äkta FPLC (GE Healthcare) and pre-equilibrated with 50 mM phosphate (pH 8), 300 mM NaCl, 10 mM EDTA and 5 mM DTT. The elution fractions containing SCO3205 were pooled and concentrated to 7 mg ml^−^^1^ and stored in aliquots at −80°C until required. The yield of SCO3205 was ∼10 mg l^−^^1^ of culture and was judged to be greater than 95% pure by SDS–PAGE analysis. The N-terminal His tag was not removed from the protein sample for any of the subsequent experiments. The protein was shown to be homodimeric, consistent with other MFRs (see Supplementary Methods) (8).

### Bioinformatics predictions of SCO3205 operator sites

**Figure 1.**
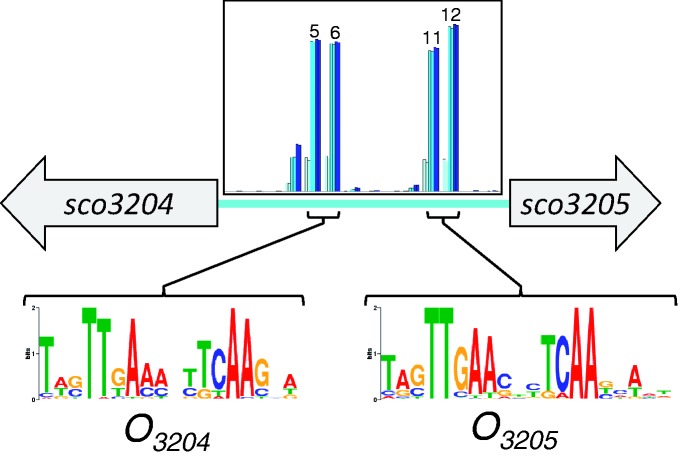
Potential operator sites for SCO3205 predicted using MEME based on a comparison with the upstream intergenic regions of orthologous genes from other actinomycetes. The left-hand motif is 36 nt upstream of *sco3204* (annotated as a hypothetical protein with homology to Class III extradiol dioxygenases) and is referred to as *O_3204_*, whereas the right-hand motif is 21 nt upstream of *sco3205* and is referred to as *O_3205_*. Also shown inset are the normalized SPR responses for the binding of SCO3205 to fragments of the *sco3204-sco3205* intergenic region, indicating the correspondence between the predicted and experimentally determined operator sites (the ‘hit' fragments are numbered). This is shown in more detail in Supplementary Figure S3A.

At the time of this study, we had access to 112 complete actinomycete genome sequences (data not shown). Using reciprocal BLAST (16), likely orthologs of SCO3205 were predicted in 62 of these genomes. Subsequently, the upstream sequences of these orthologous genes were extracted and used as input to MEME (17), searching for recurring patterns of between 10 and 25 nt in length [from the biologically relevant crystal structures of MFR–DNA complexes (1,5,18), we expected footprints of ∼22 bp]. This analysis revealed two distinct yet homologous pseudopalindromic motifs upstream of the *sco3205* coding sequence (Figure 1). The placement of these sequences suggested that SCO3205 regulates both its own transcription and that of the divergently transcribed *sco3204*, which encodes a hypothetical protein with similarity to Class III extradiol dioxygenases (http://www.ncbi.nlm.nih.gov/protein/21221639). A syntenous arrangement is apparent in 15 of the actinomycete genomes in our database. For the purposes of the ensuing discussion, we will refer to these as *O_3205_* and *O_3204_*, denoting putative SCO3205 operator sites for *sco3205* and *sco3204*, respectively. The two motifs identified by MEME were then used to search the *S. coelicolor* genome for other possible SCO3205 operator sequences using the program MAST (19), which found 27 sequences with expect values in the range 10^−^^10^ to 10^−^^7^ using the *O_3204_* motif, and 18 sequences with expect values in the range 10^−^^14^ to 10^−^^7^ using the *O_3205_* motif (Supplementary Data Set S1). Selected hits are shown in Supplementary Table S1.

### SPR experiments

**Figure 2.**
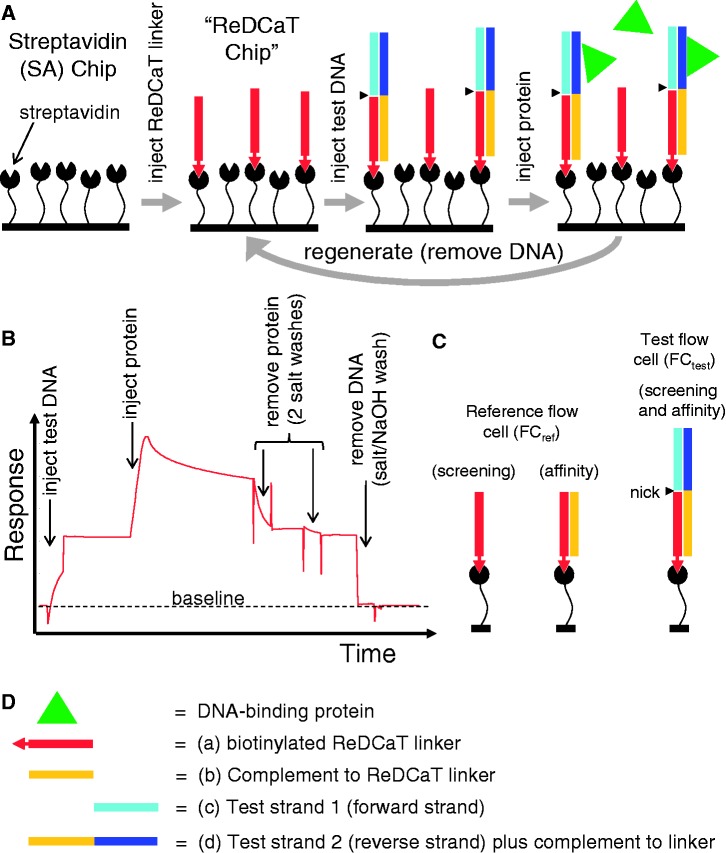
The ReDCaT methodology. (**A**) Procedure for creating, using and regenerating the ReDCaT Chip, specifically illustrating the events taking place in the test flow cell. (**B**) A typical sensorgram for the test flow cell of the ReDCaT Chip showing the responses observed during its use and regeneration. The response returns to the original baseline after stripping off the test DNA. (**C**) The composition of bound DNA in the reference and test flow cells before injecting protein in the ReDCaT screening and affinity experiments. (**D**) Key to the macromolecular components illustrated in the other three panels.

DNA samples were purchased from Sigma-Aldrich as desalted ss oligomers at 100 μM concentration in water. SPR measurements were recorded at 20°C with a Biacore T200 system (GE Healthcare). All experiments described herein were performed using ReDCaT with a single Sensor Chip SA (GE Healthcare), which has four flow cells each containing SA pre-immobilized to a carboxymethylated dextran matrix. Two types of protocol were followed (detailed in Supplementary Methods): ‘affinity’ protocols were used to determine the dissociation constants for specific protein–DNA interactions, and ‘screening’ protocols were used for all other types of experiment. For the screening experiments, flow cells 1 and 2 were used as the reference (FC_ref_) and test flow cells (FC_test_), respectively. For the affinity experiments, flow cells 3 and 4 were used as FC_ref_ and FC_test_, respectively. Throughout the SPR procedures, all samples were prepared, and all experiments were performed, in HBS-EP+ buffer [150 mM NaCl, 3 mM EDTA, 0.05% (v/v) surfactant P20, 10 mM HEPES (pH 7.4); GE Healthcare]. A cartoon representation of the ReDCaT Chip methodology and a typical SPR sensorgram are shown in Figure 2.

### Screening the *sco3204-sco3205* intergenic region for SCO3205 operator sites using SPR

**Figure 3.**
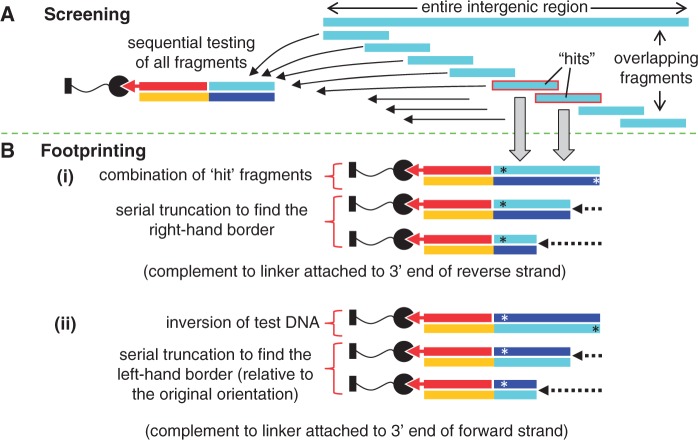
Screening for and footprinting operator sites using ReDCaT. (**A**) Screening: procedure for locating potential operator sites by testing a series of overlapping DNA fragments. (**B**) Footprinting: procedure for determining (i) the RH and (ii) the LH borders of the operator site, where the asterisks denote the 5′ ends of the starting test DNA oligomers. The same colour scheme is adopted as for Figure 2.

These experiments were performed assuming no knowledge of the bioinformatics predictions for the SCO3205 operator sites. The 119 nt sequence of the intergenic region between *sco3204* and *sco3205* in the *S. coelicolor* genome was divided into a series of overlapping fragments using the program POOP (Supplementary Program 1 and Supplementary Figure S2). Overlaps are necessary to ensure that all potential binding sites are fully present in at least one of the fragments; based on the footprints observed in the known MFR–DNA structures, this was chosen as 22 nt (1,5,18). The choice of fragment length is a compromise between the lack of resolution if larger fragments are used, and the number of SPR experiments required if shorter fragments are used. For these experiments, we chose a fragment length of 29 nt, which resulted in 14 overlapping sequences (Supplementary Figure S2 and Supplementary Table S2). For each fragment, FC_test_ was loaded with the 29 bp test DNA plus the 20 nt overhang for annealing to the linker (Figure 3A), and this gave responses of ∼400 response units (RU) (Supplementary Table S3). The responses resulting from the interaction between SCO3205 and each of the fragments were then recorded in duplicate at three different protein dimer concentrations (10, 50 and 100 nM). Negligible protein binding to FC_ref_ was observed in all experiments, with the response not exceeding 11 RU (data not shown). The level of protein binding to FC_test_ was measured 10 s after the end of the injection, and then expressed as a percentage of the theoretical maximum response, R_max_, assuming a single SCO3205 dimer binding to one immobilized ds DNA oligomer (see Supplementary Methods and Supplementary Table S3). This normalization process enabled the various responses to be readily compared, irrespective of the quantity and length of the DNA captured. ‘Hits’ were considered to be fragments that gave ∼100% of the theoretical R_max_ value at 100 nM SCO3205.

### Footprinting putative SCO3205 operator sites using SPR

The hits from intergenic screening were used to generate new oligomers as starting points to define the protein footprints of putative SCO3205 operator sites. Adjacent hits were combined into longer oligomers and captured using ReDCaT (Figure 3A). To determine the right-hand (RH) boundary of the site (defined relative to the forward strand), a series of progressively shorter DNA oligomers were synthesized, each being truncated by two nt from the 3′ end relative to the previous one, and the complementary sequence of the test DNA was truncated by the corresponding amount from its 5′ end (Figure 3B and Supplementary Tables S4 and S6). Each of the resultant footprinting oligomers was tested for SCO3205 binding. To define the left-hand (LH) boundary of the site, the same starting oligomer was used, but this was effectively inverted on the ReDCaT Chip by adding the linker to the 3′ end of the forward strand instead of the reverse strand. This resulted in placing the original LH boundary at the opposite end to the linker (Figure 3B). As before, a series of progressively shorter DNA oligomers were synthesized, each being truncated by 2 nt relative to the previous one (Supplementary Tables S4 and S6). However, this time, the truncations were made from 5′ end of the forward strand (now bearing the linker at its 3′ end) and the 3′ end of the reverse strand.

### Measuring the affinity of SCO3205 for operator sites using SPR

The affinity of SCO3205 for the operator sites was investigated using test DNA corresponding to the footprints determined earlier in the text (Supplementary Table S8). For each site, ∼90 RU of test DNA was captured at the start of each cycle. Measurements were taken in triplicate for a range of protein concentrations spanning either side of the expected *K*_D_ (0.39, 0.78, 1.56, 3.13, 6.25, 12.5, 25 and 50 nM). The inclusion of buffer-only controls enabled the use of double referencing whereby for each analyte measurement, in addition to subtracting the response in FC_ref_ from the response in FC_test_, a further buffer-only subtraction was made to correct for bulk refractive index changes or machine effects (20). SCO3205 displayed clear concentration-dependent responses for each site, and steady-state was achieved for all curves (Supplementary Figure S6).

### Prediction and testing of additional putative SCO3205 regulon members using SPR

From a comparison of the operator sites upstream of *sco3205*, a consensus motif was defined, which was used to search the *S. coelicolor* genome for other potential operator sites. For each of these matches, test DNA sequences, centred on the consensus motif, were synthesized (Supplementary Table S9). Together with one of the known sites, these new sites were tested for binding with SCO3205 (at 10, 50 and 100 nM protein) using ReDCaT. In addition to recording the responses due to protein binding, the levels of protein still bound to the DNA after the dissociation phase, and after each of two salt washes, were recorded.

### Evaluating the importance of bases in the SCO3205 consensus-binding motif using SPR

Using the same procedure as for testing the putative regulon members, each conserved nucleotide in the consensus sequence within one of the operators was replaced in turn with all three possible alternatives (Supplementary Table S12), and the binding of SCO3205 evaluated. To reduce the number of cycles necessary, only a single SCO3205 concentration of 50 nM was used for each sequence (in the previous experiment, this concentration was sufficient to obtain 100% of the theoretical R_max_ with the wild-type sequence). As an internal control, together with the substitutions at each position, the wild-type sequence was also re-analysed (Supplementary Table S13). The resultant eight replicates with the wild-type sequence were consistent, giving 98.6 ± 0.4% of the theoretical R_max_ for SCO3205 binding (Supplementary Figure S8A). After dissociation, 82.3 ± 0.2% of the protein was still bound, whereas 66.8 ± 0.4 and 60.5 ± 0.4% remained after the two salt washes, respectively (Supplementary Figure S8B).

### Protein crystallization and structure determination

The DNA for crystallization was purchased from Sigma-Aldrich as desalted ss oligomers. Each DNA oligomer was dissolved in water to obtain a 4 mM stock. Equal volumes of each complementary strand were then mixed and annealed by heating to 95°C for 10 min before cooling to 20°C. This gave ds DNA stocks at 2 mM. Several different DNA sequences and lengths were screened for crystallization based on the SPR footprints (data not shown). SCO3205 was purified as described for the SPR experiments, but L-arginine was added to the purified protein to a final concentration of 300 mM. SCO3205 was mixed with the DNA in a ratio of 1 protein dimer to 1.5 ds oligomer, and the final concentration of the SCO3205 dimer was ∼190 μM (∼8 mg ml^−^^1^).

Crystallization trials of SCO3205 with DNA were set-up using an OrxyNano robot (Douglas Instruments Ltd) in sitting-drop vapour diffusion format with 96-well MRC plates (Molecular Dimensions) using a variety of commercially available screens (Hampton Research and Molecular Dimensions) at a constant temperature of 18°C. Drops consisted of 0.3 µl of protein solution mixed with 0.3 µl of precipitant solution with a reservoir volume of 50 μl. Several conditions produced crystals, which were then optimized in a 24-well hanging-drop vapour diffusion format using XRL plates (Molecular Dimensions) with a reservoir volume of 1 ml, and drops consisting of 1 µl of protein and 1 µl of precipitant. Large single crystals (with approximate dimensions of 400 × 300 × 80 µm) appeared within 24 h in a precipitant solution consisting of 20–40% (v/v) 2-methyl-2,4-pentanediol, 80–100 mM sodium acetate and 50 mM 2-(*N*-morpholino)ethanesulfonic acid (pH 5.6). Only the drops containing 22-mer ds blunt-ended oligomers produced crystals, and, of these, only one DNA sequence yielded good quality diffraction data.

Crystals were mounted for X-ray data collection using LithoLoops (Molecular Dimensions) and then flash-cooled by plunging into liquid nitrogen and stored in Unipuck cassettes before transport to the synchrotron. A single crystal was subsequently transferred robotically to the goniostat on station I24 at the Diamond Light Source (Oxfordshire, UK), maintained at −173°C with a Cryojet cryocooler (Oxford Instruments), and diffraction data were recorded using a Pilatus 6 M detector (Dectris). The resultant images were processed using the XIA2 expert system (21–24) to 2.8 Å resolution. Analysis in POINTLESS (24) suggested that the space group was either P6_1_22 or P6_5_22, although statistical tests in TRUNCATE (25) indicated that the crystal was hemihedrally twinned (operator: k, h, −l) and must therefore belong to a lower symmetry space group, i.e. P6_1_ or P6_5_. X-ray data collection statistics are summarized in Supplementary Table S14.

SCO3205 shares 43% amino acid sequence identity with another MFR from *S. coelicolor* called AbsC (SCO5405), for which we have previously determined the X-ray crystal structure [PDB accession code 3ZMD; (26); Stevenson *et al.*, unpublished work]. A poly-Ala model of the SCO3205 protomer was generated from the AbsC structure using CHAINSAW (27). Then, an idealized B-form DNA model of the 22-mer oligomer used for crystallization was created in COOT (28). Subsequently, a molecular replacement template for the whole SCO3205-DNA complex (i.e. one protein dimer bound to one ds DNA oligomer) was generated by superposition of the individual components onto the structure of another MFR–DNA complex, namely, that of the OhrR–*ohrA* complex (PDB accession code 1Z9C) (1), using LSQKAB (29). MOLREP (30) was successful in placing two copies of this complex in the asymmetric unit (ASU), which corresponded to a solvent content of 67%, and confirmed the correct space group as P6_5_. This model was rebuilt and completed through several iterations of intensity-based twin refinement (with local non-crystallographic symmetry restraints) in REFMAC5 (31) and manual adjustment in COOT. In addition to protein, nucleic acid and solvent residues, four free phosphate groups were modelled into the density. Residual positive difference electron density was associated with the Sγ atoms of all four copies of Cys93, indicative of potential oxidation, but this was not modelled. In the latter stages, TLS refinement was used in REFMAC5 with a total of 36 TLS domains, which were defined using the TLS motion determination server (http://skuld.bmsc.washington.edu/∼tlsmd/) (32), to give final *R*_work_ and *R*_free_ values of 0.176 and 0.196 to 2.8 Å resolution, respectively. Refinement statistics are summarized in Supplementary Table S14. All structural figures were prepared using CCP4MG (33).

## RESULTS AND DISCUSSION

### Characterization of SCO3205 operator sites in the *sco3204-sco3205* intergenic region

**Figure 4.**
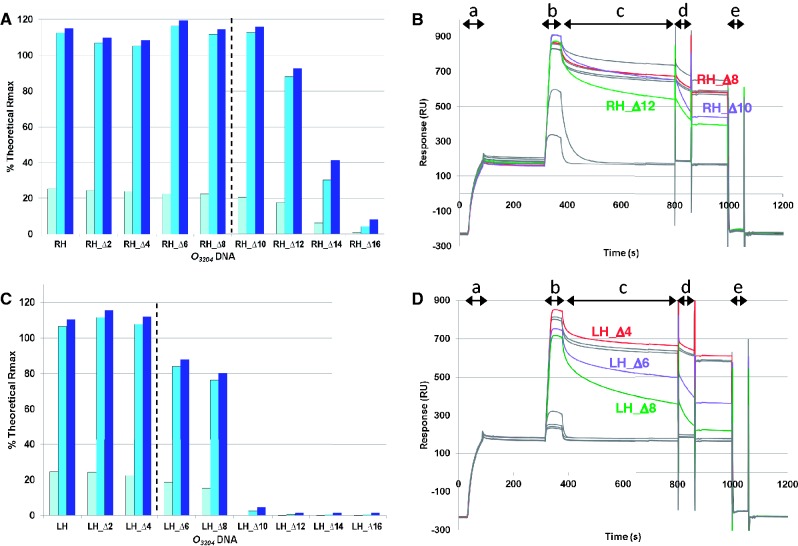
SPR footprinting of *O_3204_*. Serially truncated oligomers were used to define the extent of the *O_3204_*-binding site. Panels (**A**) and (**B**) display the data for defining the RH border, and panels (**C**) and (**D**) display the data for defining the LH border. Panels (A) and (C) show the normalized responses for each test oligomer at SCO3205 concentrations of 10 nM (pale blue), 50 nM (mid blue) and 100 nM (dark blue). The vertical dashed lines indicate the proposed footprint boundaries. Panels (B) and (D) show the corresponding sensorgrams for 100 nM SCO3205 only. The complete ‘ReDCaT cycle’ is shown for each, where: a = test DNA capture, b = protein binding, c = protein dissociation, d = salt wash, e = stripping of test DNA. Key sensorgrams are coloured, where red represents the one chosen to represent the footprint boundary, and purple and green represent the next two truncations, respectively. The remaining sensorgrams are coloured grey. N.B. No correction has been made to account for the length or quantity of test DNA captured. Thus, the maximum responses in the sensorgrams are not directly comparable. The equivalent data for *O_3205_* are displayed in Supplementary Figure S4.

A plot of the normalized SPR responses for SCO3205 binding to the screening fragments revealed two clear non-overlapping peaks, indicative of two independent binding sites in the *sco3204-3205* intergenic region (Figure 1 and Supplementary Figure S3A). These coincided with the motifs for *O_3205_* and *O_3204_* that were predicted using MEME. The responses indicated that fragments 5 and 6 were both likely to contain the complete *O_3204_* site. Thus, the starting DNA oligomer for *O_3204_* footprinting extended from the 5′ end of fragment 5 to the 3′ end of fragment 6. This corresponded to a length of 36 nt with forward sequence: 5′-ACTCCAATACTTGAACTCTCAATCTTTACGTGCCGT-3′. The experiments to determine the RH border of the site showed that removal of 10 bp (*O_3204_*_RH_Δ10) had little effect on the normalized maximum response (Supplementary Table S5 and Figure 4A). However, further truncations gave rise to progressively weaker binding, suggesting that the RH boundary of the *O_3204_* site had been crossed. Although this method of analysis is convenient for comparing the maximum responses, and thereby providing a guide to the location of the RH boundary, the complete picture was only apparent when the original sensorgrams were analysed (Figure 4B). Inspection of these revealed that, although *O_3204_*_RH_Δ10 gave a similar maximum normalized response to *O_3204_*_RH_Δ8, protein dissociation was marginally faster, and the salt wash was noticeably more effective at removing the protein from the *O_3204_*_RH_Δ10, suggesting that this oligomer was lacking part of the site. We thus selected *O_3204_*_RH_Δ8 as defining the RH boundary of the *O_3204_* site at a resolution of 2 bp.

The experiments to determine the LH border of the site showed that truncating beyond *O_3204_*_LH_Δ4 adversely affected the normalized maximum response (Supplementary Table S5 and Figure 4C), although the behaviour of this oligomer in the dissociation phase and the salt wash was comparable with that of the original 36 mer (Figure 4D). Therefore, the *O_3204_*_LH_Δ4 was used to define the LH boundary of the *O_3204_* site at a resolution of 2 bp. Thus, the *O_3204_* site footprint was determined as a 24 mer of forward sequence 5′-CAATACTTGAACTCTCAATCTTTA-3′, where the motif predicted by MEME is underlined (Supplementary Figure S5).

To determine the *O_3205_* footprint, screening fragments 11 and 12 were merged to give a starting 36 mer of forward sequence 5′-ACGCCGATTTTGTTTAATGTTCAAGGAACCGTCTCG-3′. In the experiments to define the RH boundary, *O_3205_*_RH_Δ2, *O_3205_*_RH_Δ4 and *O_3205_*_RH_Δ6 gave comparable responses and dissociation/salt wash behaviours, whereas *O_3205_*_RH_Δ8 gave a lower normalized maximum response, faster dissociation rate and was readily removed by the salt wash (Supplementary Tables S6 and S7 and Supplementary Figure S4A and B). Therefore, *O_3205_*_RH_Δ6 was chosen to define the RH boundary of the *O_3205_* site at a resolution of 2 bp. Based on the same criteria, *O_3205_*_LH_Δ6 was chosen to define the LH boundary of the *O_3205_* site (Supplementary Tables S6 and S7 and Supplementary Figure S4C and D). Thus, the *O_3205_* site footprint was determined as a 24 mer of forward sequence 5′-ATTTTGTTTAATGTTCAAGGAACC-3′, where the motif predicted by MEME is underlined (Supplementary Figure S5).

**Figure 5.**
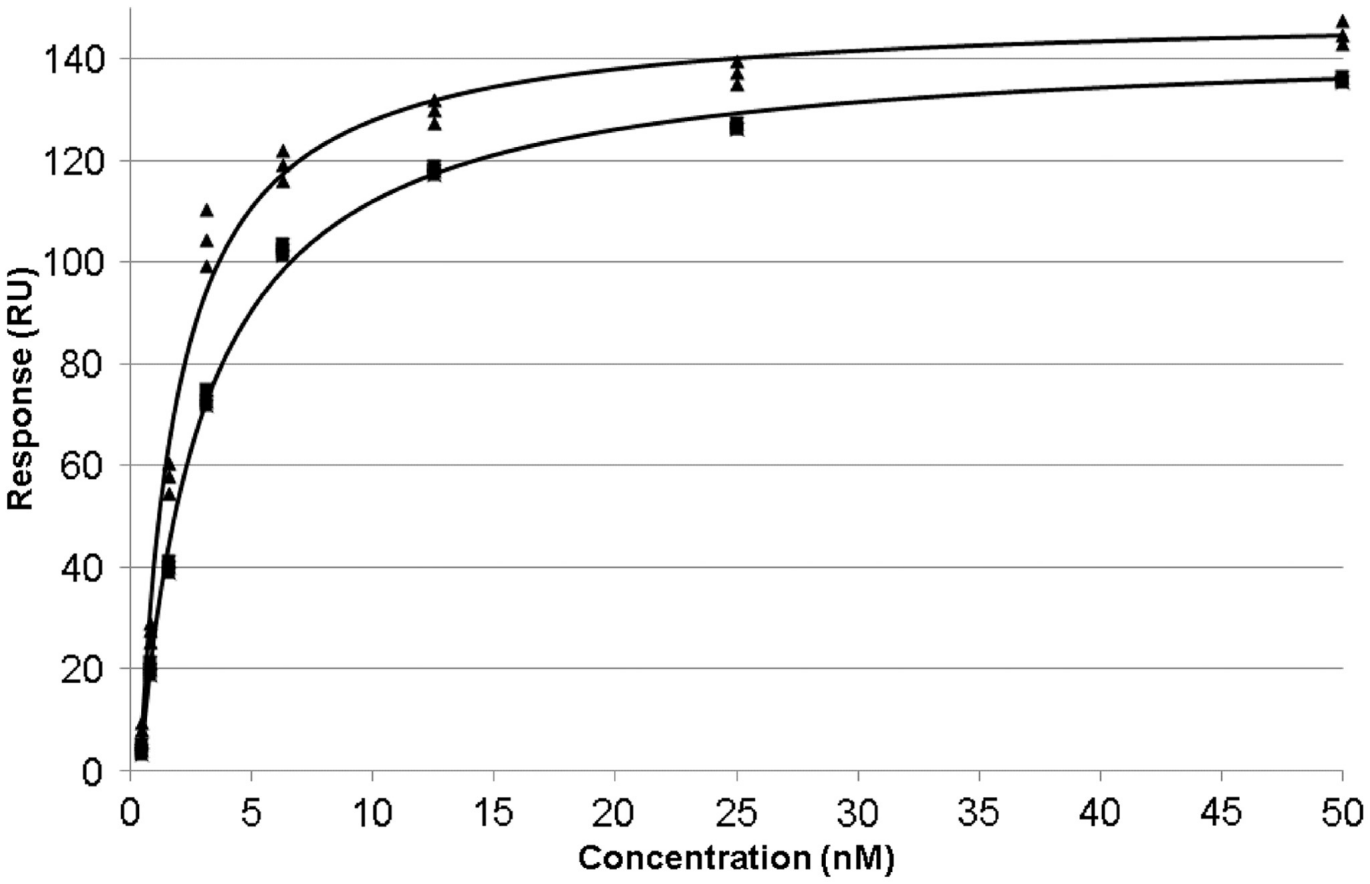
Affinity fits for SCO3205 binding to *O_3204_* and *O_3205_*. The sensorgrams obtained from the kinetic measurements using 24-mer test oligomers (Supplementary Table S8 and Supplementary Figure S6) were used to calculate the *K*_D_ values for SCO3205 binding to *O_3204_* and *O_3205_*. At each protein concentration, the responses were recorded in triplicate (4 s before the end of the injection). The results for *O_3204_* are plotted as triangles and those for *O_3205_* as squares.

The *K*_D_ values for SCO3205 binding to these 24-mer footprints (Supplementary Table S8) were calculated by fitting the responses observed at a range of protein concentrations (Supplementary Figure S6) to a steady-state affinity model assuming 1:1 binding (Figure 5), which gave values of 1.3 ± 0.2 nM and 2.4 ± 0.1 nM for *O_3204_* and *O_3205_*, respectively. These figures are comparable with those estimated for other MFRs from electrophoretic mobility shift assays (34–36).

### Prediction and testing of putative SCO3205 regulon members using SPR

**Figure 6.**
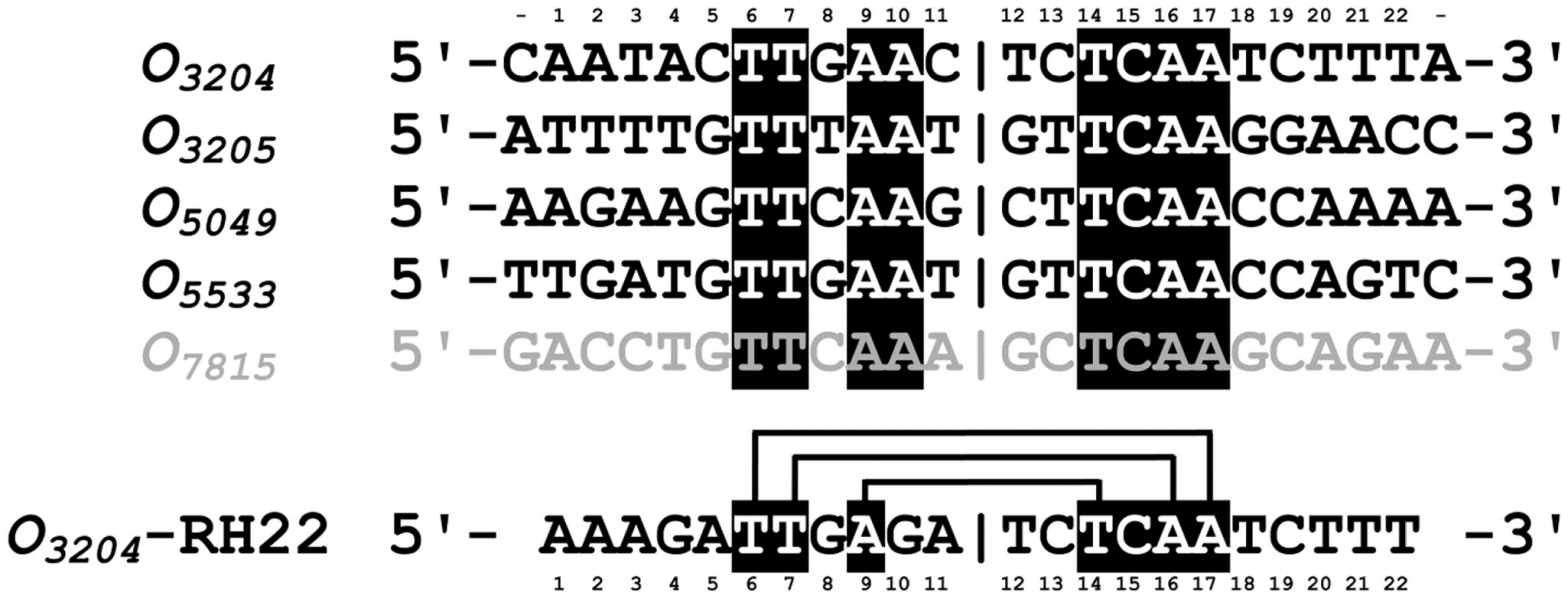
Comparison of *O_3204_* and *O_3205_* sequences with the three other sequences in the *S. coelicolor* genome that share the consensus motif highlighted with black shading. For convenience, these new sequences are named as putative operators of the nearest downstream gene. Thus, *O_5049_* is 80 nt upstream of *sco5049* (annotated as a hypothetical protein with homology to malonic semialdehyde reductase), *O_5533_* is 20 nt upstream of *sco5533* (annotated as a hypothetical protein with homology to a base-induced periplasmic protein that binds a polyisoprenoid) and *O_7815_* is 1750 nt upstream of *sco7815* (annotated as a putative TetR-family transcriptional regulator). However, the remoteness of *O_7815_* from *sco7815* suggests that it is unlikely to be an operator site for this gene; for this reason, it is shown in grey to distinguish from the other sites, which are likely to be genuine operators for SCO3205. Also shown is the 22-mer sequence used to determine the crystal structure of a SCO3205-DNA complex. The latter is a symmetrized version of *O_3204_*, where the RH half corresponds to the wild-type *O_3204_* sequence and the LH half is the reverse complement of the RH half. The numbering scheme relates to this 22 mer and is used throughout; the black lines connect symmetry-related positions within the consensus motif.

An alignment of *O_3204_* and *O_3205_* revealed the following consensus sequence: TTnAAnnnTCAA (Figure 6). In addition to *O_3204_* and *O_3205_*, three further sequences were identified containing this motif in the *S. coelicolor* genome. Two of these sites lay between pairs of divergently transcribed genes, and shall be referred to as *O_5533_* and *O_5049_*, respectively, to reflect their closest downstream genes. The third site lay 1750 nt upstream of *sco7815*. Therefore, this was most likely not a functional operator site, but for the purposes of the subsequent discussion, this will still be referred to as *O_7815_*. The SPR results showed that *O_3204_*, *O_5533_* and *O_5049_* all gave roughly 100% of the theoretical R_max_ with the highest protein concentration, whereas *O_7815_* gave roughly 50% of the theoretical R_max_ (Supplementary Table S10 and Supplementary Figure S7A). For both *O_3204_* and *O_5533_*, <20% of the total bound protein was lost during the dissociation phase, and the remaining bound protein was somewhat resistant to the salt washes, with roughly a further 20% being lost after each. For *O_5049_*, significantly more protein was lost during the dissociation phase, and the first salt wash was sufficient to remove virtually all of the remaining bound protein. By contrast, the *O_7815_* DNA was essentially stripped of all SCO3205 protein during the dissociation phase (Supplementary Table S11 and Supplementary Figure S7B).

It is notable that both *O_5533_* and *O_5049_* had been predicted by the preliminary bioinformatics analysis, whereast *O_7815_* had not (Supplementary Table S1 and Supplementary Data Set S1). We conclude that, together with *O_3204_* and *O_3205_*, *O_5533_* and *O_5049_* are likely to represent genuine operator sites for SCO3205, whereas *O_7815_* is not.

When the base preferences in the consensus sequence within *O_3204_* were tested, all of the modified sequences were still able to bind SCO3205, with none showing <70% of the theoretical R_max_ (Supplementary Table S13 and Supplementary Figure S8A). More dramatic results were apparent when the dissociation phases and salt washes were compared, especially at positions 7, 9, 14 and 16 (numbered relative to the 22 mer used to determine the crystal structure; see Figure 6), where in all cases, the most severe effect was seen when the wild-type nucleotide was substituted by its closest structural equivalent (Supplementary Figure S8B). These were either T to C or A to G changes with the net result that A:T bps were replaced with G:C bps at these positions. In all cases, a single salt wash was sufficient to remove any protein remaining after the dissociation phase. It is notable that within the *O_3204_* pseudopalindrome, position 7 of the consensus motif and the complement to position 16 are symmetry-equivalent positions (Figure 6), as are 9 and the complement to 14. At symmetry-equivalent positions 6 and 17, all substitutions had comparable deleterious effects, but these were less severe than the T to C or A to G changes at positions 7, 9, 14 and 16. Also noteworthy was the observation that position 15 was tolerant to a C to A change, but not to any other substitution. This was the only change to the consensus motif that gave near wild-type behaviour.

### Overall structure of the SCO3205-DNA complex

**Figure 7.**
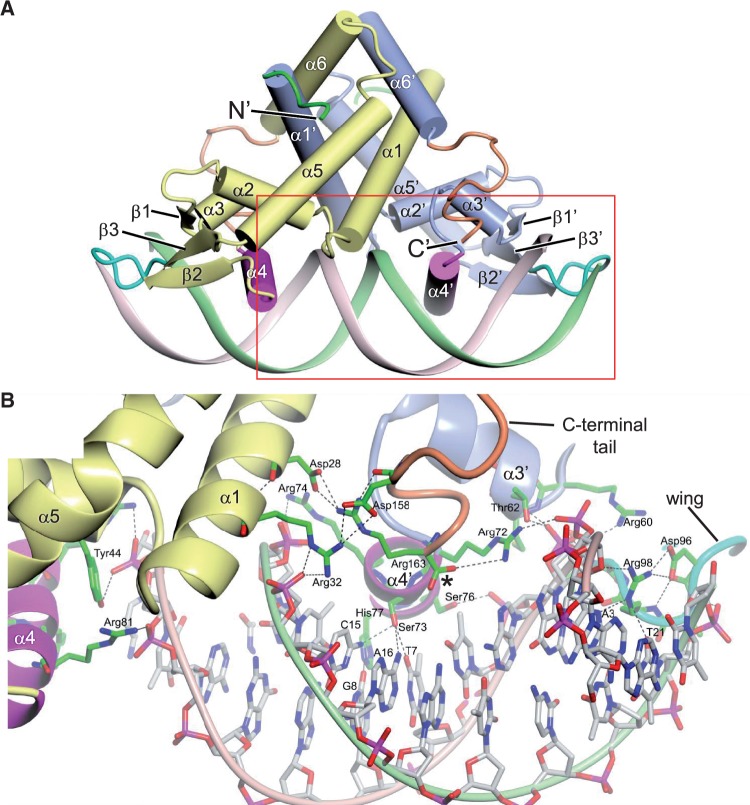
X-ray structure of the SCO3205-DNA complex. (**A**) Cartoon representation of SCO3205 bound to a 22-mer ds oligomer (which is a symmetrized version of the O*_3204_* operator; see Figure 6), with key features labelled. The two subunits are distinguished by slate blue and yellow colouration. The recognition helices are shown in magenta, the wings are shown in cyan and the N- and C-termini are shown in green and orange, respectively. (**B**) Detail of the region bounded by the red rectangle in panel (A). Shown as dashed lines are the key protein–DNA interactions involving the recognition helices, the wing and the C-terminal tail (also see Supplementary Figure S12). The C-terminal carboxyl group is indicated by the asterisk. For clarity, the base-pairing hydrogen bonds have been omitted.

Guided by the SPR footprinting, we were able to define oligomers suitable for co-crystallization with SCO3205. The resultant crystal structure reveals two SCO3205 homodimers in the ASU, each bound to its own ds 22-mer DNA. The ends of the latter make base-to-base stacking interactions with equivalent 22 mers in adjacent unit cells to form pseudo-continuous double-helical DNA filaments that permeate the crystal. The SCO3205 homodimers are closely similar (root mean square deviation based on Cα atoms = 0.16 Å) and adopt the typical MFR overall triangular shape (Figure 7A). Each subunit is made up of six α-helices, three 3_10_-helices and a three-stranded, antiparallel β-sheet (Supplementary Figure S9), which fold into two domains. The dimerization domain constitutes helices α1, α5 and α6, where α1 inserts between α5′ and α6′ of the opposing subunit, and vice versa, to generate a dimer interface covering on average 2660 Å^2^ and representing 24% of the total solvent accessible surface per subunit (as calculated using the PISA server; http://www.ebi.ac.uk/msd-srv/prot_int) (37). The DNA-binding domain contains helices α2, α3 and α4 and the β-sheet and includes a winged helix DNA-binding motif (38), where α3 and α4 form a classical helix-turn-helix, and the loop delineated by β2 and β3 corresponds to the ‘wing’ (Figure 7A).

### Comparison of SCO3205 with other MFR–DNA structures

Interrogation of the Protein Data Bank using the DALI server (http://ekhidna.biocenter.helsinki.fi/dali_server) (39) retrieves 51 non-redundant entries with high structural similarity to SCO3205 at the level of the subunit (*Z*-scores > 10.0). Amongst these are the three biologically relevant MFR–DNA complex structures, namely, *Bacillus subtilis* OhrR (PDB code: 1Z9C, *Z*-score = 16.3) (1), *Salmonella enterica* SlyA (PDB code: 3Q5F, *Z*-score = 16.1) (5) and *Mycobacterium tuberculosis* MosR (PDB code: 4FX4, *Z*-score = 15.4) (18), which are broadly similar to the SCO3205-DNA complex (Supplementary Table S15 and Supplementary Figures S10 and S11), and, although the conformation of the DNA is somewhat variable, in none of the complexes is the DNA significantly bent.

The native sequence of SCO3205, at 163 residues, is 16 amino acids longer than OhrR, 20 longer than SlyA and 11 longer than MosR. Structure-based sequence alignments (data not shown) indicate that SCO3205 is lengthened at both the N- and C-termini relative to these other proteins, and this is evident in a superposition of their subunits (Supplementary Figure S11). Before Leu8, the N-terminus of SCO3205 adopts a variety of conformations, and, where it is ordered, it extends away from the core of the homodimer and is stabilized through contacts with neighbouring homodimers in the crystal. Significantly, the C-terminus is ordered in all four subunits in the ASU, with the C-terminal residue, Arg163, clearly visible in electron density. No such C-terminal tail is present in the three other structures.

**Figure 8.**
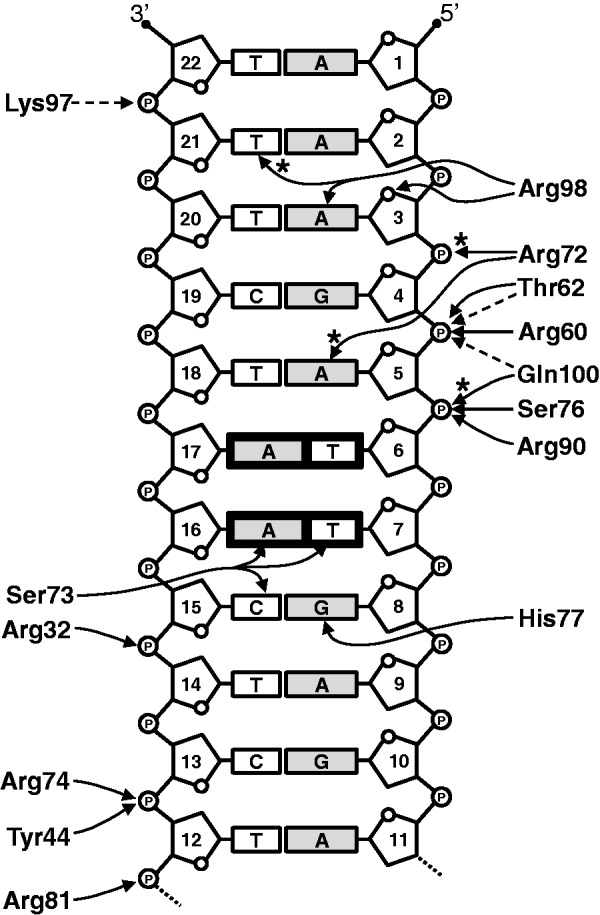
Schematic diagram summarizing the hydrogen bonds and salt bridges at the protein–DNA interface of the SCO3205-DNA complex. Owing to the symmetric nature of the DNA, only half of the ds 22-mer is depicted. Solid lines indicate side-chain interactions, whereas dashed lines indicate those involving the protein main-chain. Interactions not present in every interface are marked with an asterisk. The black boxes highlight the TT/AA palindromic sequence that is prominent in putative SCO3205 operator sites.

The SCO3205 dimer makes extensive contacts with the DNA duplex (Figures 7B and 8 and Supplementary Figure S12) spanning some 1920 Å^2^ (calculated using PISA), and the majority of the interactions fall into three types. Those involving the helix-turn-helix motif, those involving the wing, and those involving the C-terminal tail. These will be discussed below and, where appropriate, interpreted in the context of the SPR data.

### DNA interactions involving the helix-turn-helix motif of SCO3205

The recognition helices (α4 and α4′) engage with consecutive major grooves and interact with both the bases and the phosphate backbone (Figure 7 and Supplementary Figure S12A). Contacts with DNA bases in the major groove are made by just three residues: Arg72, Ser73 and His77. In one subunit from each homodimer in the ASU, Arg72 donates a hydrogen bond via the Nη^1^ atom of its guanidinium group to N^7^ of A5, whereas the Nη^2^ atom of its symmetry mate in the opposing subunit forms a salt bridge with the phosphate group of G4. Ser73 Oγ donates a hydrogen bond to O^4^ of T7 and accepts hydrogen bonds from both N^4^ of C15 and N^6^ of A16, whereas His77 Nε^2^ donates a hydrogen bond to O^6^ of G8 (Figure 8). The SPR experiments showed that a C15A substitution was tolerated well, giving wild-type like behaviour. Although this represents a pyrimidine to purine change, at position 15, this would place the N^6^ of adenine roughly where the N^4^ of cytosine was, and at position 8, this would place the O^4^ of thymine roughly where the O^6^ of guanine was. Thus, with minor side-chain adjustments, Ser73 and His77 could conceivably hydrogen bond to these bases in a similar fashion to those in the wild-type sequence. Three further residues from the recognition helix make contacts exclusively to the phosphate backbone: the guanidinium groups of Arg74 and Arg81 form salt bridges with the phosphates of C13 and T12, respectively, and Ser76 Oγ donates a hydrogen bond to the phosphate of T6.

The major groove of the DNA is markedly widened to accommodate the recognition helices (Supplementary Figure S13). This is maximal at the invariant T6:A17 and T7:A16 bps. Given the increased flexibility of A:T bps over G:C bps, these bases may have been conserved because they permit this major groove widening and thus could play a role in indirect readout of the DNA sequence (40). Consistent with this hypothesis, the SPR experiments showed that all substitutions at T6:A17 and T7:A16 had deleterious effects, especially T7C and A16G. Although the latter substitutions would also alter the direct interactions with Ser73, the lack of direct protein contacts to T6:A17 would support an indirect readout function for at least this bp. By contrast, the major groove is significantly narrowed at the invariant A9:T14 bps (Supplementary Figure S13), where A9G and T14C substitutions have the most deleterious effects in the SPR experiments. The lack of direct protein contacts here too may also imply that it is the flexibility of an A:T pair that is important at this position.

Helix α3 also makes contact with the phosphate backbone: the guanidinium group of Arg60 forms a salt bridge with the phosphate of A5, and Thr62 donates hydrogen bonds to this same phosphate via its side-chain Oγ^1^ and its main-chain NH group (Figure 8). There is additionally a van der Waals interaction between Met 61 and the phosphate of T6. Also contributing to the DNA-binding affinity of SCO3205 is the positive dipole that exists at the N-terminus of helix α3 (41), which is directed at the phosphate backbone at A5 (Figure 7B).

### DNA interactions involving the wing of SCO3205

The wing, delineated by β2 and β3, extends across the minor groove and makes a number of contacts with the phosphate backbone (Figure 7 and Supplementary Figure S12B). Within β2, Arg90 salt bridges to the phosphate of T6 via both its Nη^2^ and Nε atoms (Figure 8). The remainder of the interactions exclusively involve the β-hairpin: hydrogen bonds are donated from the main-chain NH groups of Lys97 and Gln100, and from Nε^2^ of Gln100, to the phosphates of T22, A5 and T6, respectively (although the latter interaction is only seen for one subunit). Additionally, there are van der Waals interactions between Asp96 and Gly99 with the phosphates of T22 and A5, respectively. Perhaps, the most significant interactions involve Arg98: the electropositive guanidinium group is inserted into the minor groove (Figure 7 and Supplementary Figure S12B), where the electronegative potential of the phosphate backbone is focused (42), and it donates hydrogen bonds to both the O^4'^ of the deoxyribose moiety and N^3^ of A3 via its Nη^2^ and Nε atoms, respectively. In some subunits, the Nε atom also appears to donate to O^2^ of T21, and, therefore, it is forming a bifurcated hydrogen bond involving both of these bases. Moreover, the side-chain of Arg98 is correctly presented for engagement with the minor groove through a further bifurcated hydrogen bond to Asp96 via its Nη^1^ atom. Both the 2:21 and 3:20 bps are variable across the putative regulon members (Figure 5); presumably, the plasticity of the wing would allow the guanidinium group of Arg98 to realign and make equally favourable contacts with these alternative sequences. Indeed, the flexibility of the wing is illustrated by the observation that it is frequently disordered in crystal structures determined in the absence of DNA, unless it is stabilized by crystal contacts (43–45). The important contribution made by Arg98 to the protein–DNA interaction is illustrated by the SPR footprinting experiments, as removal of the 2:21 and 3:20 bps from either end of both *O_3204_* and *O_3205_* results in a reduction in the normalized maximum responses due to SCO3205 binding and an increase in the protein dissociation rates (see results for *O_3204_*_RH_Δ12, *O_3204_*_LH_Δ8, *O_3205_*_RH_Δ10 and *O_3205_*_LH_Δ10 in Figure 4 and Supplementary Figure S4).

### DNA interactions involving the C-terminal tail of SCO3205

The C-terminal extension of SCO3205 also plays a role in stabilizing the protein–DNA interface, albeit indirectly. The C-terminal residue itself, Arg163, has a dual function: the side-chain anchors the tail to the core of the homodimer via salt bridges to Asp28 in helix α1, whereas via a further salt bridge, the carboxy terminus orients the guanidinium group of Arg72 (in the recognition helix) for interaction with either N^7^ of A5, or the phosphate group of G4. Also in the tail, Asp158, salt bridges to Nη^1^ of Arg32 in helix α1, which in turn salt bridges to the phosphate of C15 via both its Nη^2^ and Nε atoms (Figure 7B and Supplementary Figure S12C). A further interaction outside the aforementioned three regions involves a hydrogen bond donated by Tyr44 in helix α2 to the phosphate of C13.

### The biological role of SCO3205

Based on the currently available data, we are not able to ascribe a function to SCO3205. Despite the strong representation of likely SCO3205 orthologs across the sequenced actinomycete genomes, none of them have thus far been characterized. Moreover, although the bioinformatics analysis suggests a pleiotropic role, gene annotations for the putative regulon members do not indicate a clear physiological theme.

Although, we have yet to produce a gene knockout to probe SCO3205 function through phenotypic screening, our past experience with other uncharacterized streptomycete MFRs would suggest that this is rarely informative (data not shown). It requires the experiments to be conducted under the appropriate (but *a priori* unknown) physiological conditions to reveal a phenotype, and that the organism is not able to compensate for the deletion through the deployment of homologous genes. In relation to the latter, the *S. coelicolor* genome encodes two further MFRs that are closely related to SCO3205, namely, AbsC (SCO5405; 43% sequence identity) and SCO7727 (38% sequence identity). AbsC has been implicated in the control of zinc-dependent antibiotic production (46), whereas SCO7727 is uncharacterized. Of the 13 residues that form side-chain-mediated interactions with DNA in the SCO3205-DNA complex (Figure 8), respectively, 11 and 10 of these are either conserved or semi-conservatively substituted in AbsC and SCO7727 (data not shown), suggesting that these homologs are likely to have overlapping regulons. The original bioinformatics analysis using MAST found matches with both the *O_3204_* and *O_3205_* motifs upstream of *sco7727* (two discrete sites) and *absc* (a single site) with expect values in the range 10^−^^6^ to 10^−^^5^ (Supplementary Table S1 and Supplementary Data Set S1). Given the tendency of bacterial transcription factors to negatively autoregulate (9), these sites are also likely to be operators for SCO7727 and AbsC, respectively. Additionally, the MAST search found a match to *O_3204_* (with expect value 7.87 × 10^−^^6^) in the intergenic region between the divergently transcribed *sco7681* and *sco7682*, which corresponds to a known operator site for AbsC (46).

The molecular surface of SCO3205 presents a number of possible clefts that could accommodate a small molecule effector ligand (not shown). The most striking of these corresponds to that seen occupied by salicylate in other MFRs, including MTH313 from *Methanobacterium thermoautotrophicum* (PDB code: 3BPX) (47) and AbsC (PDB code: 3ZMD; Stevenson *et al.*, unpublished). The pocket is formed mainly by residues from helices α1, α2′ and α5′. In AbsC, Trp18 and His120, both donate hydrogen bonds to the carboxylate group of the salicylate ligand. This residue pair is conserved in both SCO7727 and SCO3205 (Trp16 and His119; SCO3205 numbering), suggesting that all three MFRs may bind similar ligands. Overall, we conclude that it is likely that the biological function of SCO3205 overlaps with those of AbsC and SCO7727.

## CONCLUSIONS

In this study, we identify regulon candidate genes for SCO3205, a MFR from *S. coelicolor*. We determine dissociation constants for the interactions between SCO3205 and two putative operators and probe the importance of individual bases in a consensus binding sequence. Through the subsequent determination of a SCO3205-DNA complex X-ray structure, we are able to interpret our data within the context of a structural framework. The placement of two SCO3205 operators within the intergenic region upstream of both *sco3205* and the divergently transcribed *sco3204* is consistent with it repressing its own transcription and that of the *sco3204* gene product, respectively; the nanomolar dissociation constants determined for these sites being comparable with those obtained for other MFRs (34–36).

We demonstrate that the combination of ReDCaT and the SPR footprinting provides a robust, quantitative and inexpensive approach to locating and defining DNA-binding sites for proteins. Not only does ReDCaT lower the cost of these experiments, it also renders them highly automatable. For example, the footprinting experiments alone required a total of 138 injections of test DNA. Using ReDCaT, this was possible in a single run, on the same chip, and without any user intervention. By contrast, if direct capture had been used, the chip would need to be replaced manually after every three injections of test DNA (assuming three flow cells are used for test DNA and one as a reference for each chip); this would be a labour-intensive experiment and would consume 46 chips. The SPR methods described in this work are entirely generic, being applicable to a range of protein–nucleic acid interactions. Moreover, they are not reliant on bioinformatics predictions of binding sites based on multiple genome sequences; all that is required is the nucleotide sequence of a region that is likely to contain a protein-binding site.

Traditional DNA footprinting methods work on the principle that the DNA-binding protein (or ligand) under investigation specifically protects the DNA from cleavage by a biological or chemical agent. The most commonly used agents being DNase I (48) and hydroxyl radicals (49). Both approaches typically use radiolabelled DNA together with gel electrophoresis to detect cleavage, although, more recently, fluorescently labelled DNA combined with capillary electrophoresis has also been successfully used (50). DNase I is a ∼30 kDa protein, and thus footprinting methods using this enzyme all suffer from a lack of accuracy due to steric clashes between the enzyme and the protein of interest, leading to an over-estimation of the footprint size (51). Moreover, DNase I does not cleave DNA non-specifically, as its activity is affected by local DNA structure and sequence, which can further exacerbate the inaccuracy of the method. On the other hand, the hydroxyl radical approach is not subject to steric or DNA sequence-dependent limitations, and thus can deliver a highly accurate footprint. However, this latter method is more experimentally demanding than DNase I footprinting and, as a result, is much less popular (51). As our SPR footprinting approach does not rely on protection of the DNA from nuclease cleavage to define protein-binding sites, it can do so with comparable accuracy with that of the hydroxyl radical method. Moreover, it offers significant advantages over the latter in terms of its simplicity.

The SPR footprinting of the *O_3204_* and *O_3205_* operators showed that, in both cases, the SCO3205 homodimer covered some 24 bp at a resolution of 2 bp, and this guided the choice of oligomers for co-crystallization. The resultant crystal structure showed that the contacts made by SCO3205 spanned the whole of the blunt-ended 22 mer that was used for co-crystallization, with hydrogen bonds donated to the last phosphate at the 3′ end of each strand. This footprint was consistent with those observed in the crystal structures of other MFR–DNA complexes (1,5,18), although in each of these, the ds oligomers used for co-crystallization were longer and sticky ended. Thus, we demonstrate that SPR footprinting is a viable, label-free, alternative to the more conventional footprinting methods, and that it is particularly useful for defining suitable oligomers for co-crystallization towards the determination of novel protein-nucleic acid complex structures.

## ACCESSION NUMBERS

Coordinates and structure factors for the SCO3205-DNA complex structure described herein have been deposited in the Protein Data Bank with accession code 3ZPL.

## SUPPLEMENTARY DATA

Supplementary Data are available at NAR Online: Supplementary Methods, Supplementary Tables 1–15, Supplementary Figures 1–13, Supplementary References [1, 5, 10, 12, 18, 45, 52–56], Supplementary Data Set 1 and Supplementary Program 1.

## FUNDING

Biotechnology and Biological Sciences Research Council grant [BB/J004561/1] (to the John Innes Centre); the John Innes Foundation; a Marie Curie Incoming International Fellowship (to S.G.); and a John Innes Centre Rotation Studentship (to T.B.K.L.). Funding for open access charge: BBSRC [BB/J004561/1] (to the John Innes Centre).

*Conflict of interest statement*. None declared.

## Supplementary Material

Supplementary Data
