# Supplementary material for: Investigation of DNA sequence recognition by a streptomycete MarR family transcriptional regulator through surface plasmon resonance and X-ray crystallography
Source: Nucleic Acids Res. 2013 Jun 7;41(14):7009–22. doi: 10.1093/nar/gkt523 (PMC3737563; doi:10.1093/nar/gkt523)
Supplement: Supplementary Data [file supp_41_14_7009__index.html]

Investigation of DNA sequence recognition by a streptomycete MarR family transcriptional regulator through surface plasmon resonance and X-ray crystallography — Supplementary Data 

# Investigation of DNA sequence recognition by a streptomycete MarR family transcriptional regulator through surface plasmon resonance and X-ray crystallography

## 

files

**Files in this Data Supplement:**

- Supplementary Data - pdf file
- Supplementary Data - xls file
- Supplementary Data - pl file
