# Supplementary material for: Investigation of DNA sequence recognition by a streptomycete MarR family transcriptional regulator through surface plasmon resonance and X-ray crystallography
Source: Nucleic Acids Res. 2013 Jun 7;41(14):7009–22. doi: 10.1093/nar/gkt523 (PMC3737563; doi:10.1093/nar/gkt523)
Supplement: Supplementary Data [file supp_gkt523_Stevenson_SCO3205_Supplementary_Data_FINAL.pdf]

Supplementary Data for:

Investigation of DNA sequence recognition by a streptomycete MarR family transcriptional regulator through surface plasmon resonance and X-ray crystallography

Clare E. M. Stevenson[1*], Aoun Assaad[1], Govind Chandra[2], Tung B. K. Le[2], Sandra J. Greive[1], Mervyn J. Bibb[2], and David M. Lawson[1*]

[1]Department of Biological Chemistry, John Innes Centre, Norwich Research Park, Norwich NR4 7UH, UK
[2]Department of Molecular Microbiology, John Innes Centre, Norwich Research Park, Norwich NR4 7UH, UK

* To whom correspondence should be addressed. David M Lawson, Tel: +44 1603 450725; Fax: +44 1603 450018; email: david.lawson@jic.ac.uk. Correspondence may also be addressed to Clare E. M. Stevenson, Tel: +44 1603 450734; Fax: +44 1603 450018; email: clare.stevenson@jic.ac.uk

Present addresses:

Tung B. Le, Massachusetts Institute of Technology, Department of Biology, 68-570, 77 Massachusetts Avenue, Cambridge, MA 02139. USA

Sandra J. Greive, York Structural Biology Laboratory, Department of Chemistry, University of York, York, YO10 5DD, UK

SUPPLEMENTARY METHODS

Determination of the oligomeric state of SCO3205

Dynamic Light Scattering was used to monitor the solution properties of the purified protein. For this purpose, approximately 30 µl of the sample was filtered through a 0.1 µm Ultrafree-MC filter (Millipore) to remove particulate material before introduction into a 12 µl cuvette. The cuvette was placed in a DynaPro Titan molecular sizing instrument at 20^{0}C (Wyatt Technology). A minimum of ten scattering measurements were obtained and the resulting data were analyzed using the DYNAMICS software package (Wyatt Technology). The sample exhibited a monomodal size distribution, with a polydispersity of 11.2% and a molecular-size estimate of 48.0 kDa. This was very close to the value of 47.7 kDa estimated from the gel filtration column, which had been calibrated with a Gel Filtration LMW Calibration Kit (GE Healthcare). This suggests that SCO3205 exists as a homodimer (calculated molecular mass 41.1 kDa for the His-tagged dimer), in agreement with other characterized MarR homologues.

DNA preparation for SPR experiments

Four types of DNA oligomer were used:

(a) ReDCaT linker: 20 nt 5'-biotinylated oligomer [designed to have no secondary structure or tendency to form dimers, as assessed by the Sigma-Aldrich website (www.sigma-gonosys.com/calc/DNACalc.asp); see Supplementary Table S2 for the sequence].
(b) Complement to the ReDCaT linker.
(c) Strand 1 of test DNA (forward strand by default).
(d) Strand 2 of test DNA (reverse strand by default) with the complement to the ReDCaT linker attached to the 3' end.

The test DNA samples were prepared by annealing together oligomers of type (c) and (d) using a slight excess (1.2:1) of type (c) to minimize the likelihood of free type (d) that would compete with annealed test DNA for the immobilized linker. Oligomers of type (a) and (b) were annealed to use as a reference in the affinity experiments (see below). The annealing procedure consisted of mixing the complementary oligomers and heating to 95°C for 10 min, before cooling to 20^{0}C. The various DNA oligomers were diluted to their working concentrations using HBS-EP+ buffer [150 mM NaCl, 3 mM EDTA, 0.05% (v/v) surfactant P20, 10 mM HEPES pH 7.4; GE Healthcare]. Throughout the SPR procedures, this will be referred to simply as "buffer".

SPR measurements

Preliminary experiments with SCO3205 had established that salt washes were frequently not sufficient to remove all of the bound protein from the test DNA. Thus, to ensure consistency, the test DNA was stripped and re-captured between each cycle, even if the same test DNA was to be used again. The following protocols were automated through the appropriate "wizards" in the Biacore T200 BiaEvaluation software version 1.0 (GE Healthcare).

Protocol 1 - preparation of the ReDCaT Chip for the screening experiments:

1. To remove any unconjugated streptavidin, the chip surface was washed using three injections of 1.0 M NaCl, 50 mM NaOH for 60 s, each followed by buffer for 60 s (all at 10 µl min^{-1}).
2. The ReDCaT linker (100 nM) was injected at 5 µl min^{-1} over FC_{ref} and FC_{test} to give a relatively high immobilization level (approx. 500 RU).

Protocol 2 - use of the ReDCaT Chip for the screening experiments:

The flow rate was 30 µl min^{-1} unless stated otherwise. Step 3 was optional.

1. Test DNA (1.0 µM) was injected over FC_{test} at a flow rate of 10 µl min^{-1} for 60 s, followed by buffer for 120 s.
2. Protein at the required concentration (or buffer-only control) was injected over FC_{ref} and FC_{test}, for 60 s, followed by buffer for 360 s.
3. To examine the effect of NaCl on the protein-DNA interaction, 0.5 M NaCl was injected for 60 s over FC_{ref} and FC_{test}, followed by buffer for 60 s. In some cases, the NaCl injection was repeated.
4. The test DNA (together with any remaining bound protein) was then removed by injecting 1.0 M NaCl, 50 mM NaOH over FC_{ref} and FC_{test} at a flow rate of 10 µl min^{-1} to leave only the ReDCaT linker (the response should return to the level prior to step 1). Buffer was flowed over both flow cells for a further 120 s.

Cycles 1-4 were repeated as many times as required (e.g. with different DNA and protein samples), without any further user intervention. When the experiment was completed, the chip was removed from the instrument and stored in buffer at 4°C until required again.

Protocol 3 - preparation of the ReDCaT Chip for the affinity experiments:

1. To remove any unconjugated streptavidin, the chip surface was washed using three injections of 1.0 M NaCl, 50 mM NaOH for 60 s, each followed by buffer for 60 s (all at 10 µl min^{-1}).
2. The ReDCaT linker, pre-annealed to its complement, was injected at 5 µl min^{-1} over FC_{test} to give a relatively low immobilization level (approx. 50 RU).
3. The ReDCaT linker (10 nM) was injected at 5 µl min^{-1} over FC_{ref} to give a relatively low immobilization level (approx. 130 RU).

Protocol 4 - use of the ReDCaT Chip for the affinity experiments:

The flow rate was 100 µl min^{-1} unless stated otherwise.

1. Test DNA (0.5 µM) was injected over FC_{test} at a flow rate of 10 µl min^{-1} for 30 s and the baseline was allowed to stabilize with buffer flowing for a further 60 s.
2. Protein at the required concentration (or buffer-only control) was injected over FC_{ref} and FC_{test}, for 210 s, followed by buffer for 600 s to monitor the protein dissociation.
3. The test DNA (together with any remaining bound protein) was then removed by injecting 1.0 M NaCl, 50 mM NaOH over FC_{test} at a flow rate of 10 µl min^{-1} for 60 s to leave only the ReDCaT linker (the

response should return to the level prior to step 1). Buffer was flowed over both FC_{ref} and FC_{test} for a further 90s.

Cycles 1-3 were repeated in triplicate for a range of protein concentrations spanning either side of the expected K_D (estimated from preliminary experiments). When the experiment was completed, the chip was removed from the instrument and stored in buffer at 4°C until required again.

SPR data analysis

All sensorgrams were analysed using Biacore T200 BiaEvaluation software version 1.0 (GE Healthcare). The data were then plotted using Microsoft Excel.

Normalizing responses due to protein binding in SPR experiments

In order to readily compare the results from the SPR experiments, the responses recorded due to protein binding were normalized as described below.

The theoretical maximum response, R_{max}, for SCO3205 (the "analyte") binding to the test DNA (the "ligand") was calculated using the formula:

theoretical R_{max} = (mol.mass analyte/mol.mass ligand) x (response for ligand capture) x (stoichiometry)

However, when the ligand is DNA, it has been suggested that the result needs to be multiplied by a factor of 0.78 because the response associated with nucleic acid binding to the surface is not the same as that for a protein of equivalent mass (10,12). This correction was made in all the R_{max} calculations in the present study. Moreover, all calculations were made assuming a stoichiometry of one SCO3205 dimer binding to one ds oligomer of test DNA. Thus, for this system:

theoretical R_{max} = (mol.mass SCO3205 dimer/mol.mass test DNA) x DNA captured x 1 x 0.78

Then, the percentage of R_{max} measured upon protein binding is calculated as follows:

% of R_{max} measured = (measured R/theoretical R_{max}) x 100

For example in the intergenic screening, for fragment 5, replicate 1 at 100 nM SCO3205 (data highlighted in Supplementary Tables S2 and S3):

theoretical R_{max} = (41046/23787) x 420.1 x 1 x 0.78 = **565.4 RU**

% of R_{max} measured = (655.8/565.4) x 100 = **116.0%**

This figure of greater than 100% of R_{max} could be attributed to a small amount of additional non-specific binding giving rise to more than one protein dimer binding per immobilized DNA duplex. Alternatively, it could be due to an underestimation of the theoretical R_{max}. Indeed, it has been suggested that the 0.78 correction factor is not necessary (52), which would give rise to the following adjusted values:

theoretical R_{max} = (41046/23787) x 420.1 x 1 = **724.9 RU**

% of R_{max} measured = (655.8/724.9) x 100 = **90.5%**

This would indicate that full 1:1 binding has not been achieved in this experiment. However, for the purposes of the analysis presented herein, the absolute values of these normalized responses are not crucial. Instead, it is their relative values that enable us to determine which of the intergenic screening fragments contain putative operator sites, and which of the footprinting truncated oligomers define the borders of these operator sites. Nevertheless, normalized response values approaching 200%, for example, could be indicative of specific 2:1 binding i.e. two protein dimers binding per ds DNA oligomer.

Table S1. Putative regulon members for SCO3205 predicted using MAST

Closest downstream gene	Hits using O_{3204} motif		Hits using O_{3205} motif		Gene annotation
	MAST score	expect value	MAST score	expect value	
sco3204	1699	3.58×10^{-8}	2877	1.62×10^{-14}	hypothetical protein with homology to Class III extradiol dioxygenases
sco3205	2315	2.23×10^{-10}	1132	8.59×10^{-7}	MarR family transcriptional regulator
sco5533	2186	8.07×10^{-10}	1321	3.62×10^{-7}	hypothetical protein with homology to a base-induced periplasmic protein that binds a polyisoprenoid
sco5049	1771	2.22×10^{-8}	1267	4.70×10^{-7}	hypothetical protein with homology to malonic semialdehyde reductase
sco7727	640	4.51×10^{-6}	848	2.48×10^{-6}	MarR family transcriptional regulator
sco7728	68	2.21×10^{-5}	104	1.92×10^{-5}	hypothetical protein belonging to N-acyltransferase superfamily
sco5405	425	8.53×10^{-6}	347	1.02×10^{-5}	MarR family transcriptional regulator (AbsC)
sco7681	454	7.87×10^{-6}	-	-	AMP-binding ligase from coelibactin cluster

Selected hits found by MAST using the O_{3204} and O_{3204} motifs identified by MEME. The full MAST output is given in Supplementary Dataset S1.

Table S2. Sequences of fragments used for screening the *sco3204-sco3205* intergenic region

screening oligo	length (bases)	sequence 5' to 3'	molecular mass (Da)	F+R annealed molecular mass (Da)
ReDCaT linker	20	Biotin-gcaggaggacgtagggtagg	8718	-
ReDCaT linker complement	20	cctaccctacgtcctcctgc	5925	14642
fragment_1_F	29	GCGCTCCCGGACGGCGGCGGCCATGGCTA	8925	-
fragment_1_R	49	TAGCCATGGCCGCCGCCGTCCGGGAGCGCcctaccctacgtcctcctgc	14872	23797
fragment_2_F	29	CGGACGGCGGCGGCCATGGCTACTCCAAT	8908	-
fragment_2_R	49	ATTGGAGTAGCCATGGCCGCCGCCGTCCGcctaccctacgtcctcctgc	14886	23794
fragment_3_F	29	CGGCGGCCATGGCTACTCCAATACTTGAA	8866	-
fragment_3_R	49	TTCAAGTATTGGAGTAGCCATGGCCGCCGcctaccctacgtcctcctgc	14925	23791
fragment_4_F	29	CATGGCTACTCCAATACTTGAACTCTCAA	8785	-
fragment_4_R	49	TTGAGAGTTCAAGTATTGGAGTAGCCATGcctaccctacgtcctcctgc	15003	23788
fragment_5_F	29	ACTCCAATACTTGAACTCTCAATCTTTAC	8735	-
fragment_5_R	49	GTAAAGATTGAGAGTTCAAGTATTGGAGTcctaccctacgtcctcctgc	15052	23787
fragment_6_F	29	TACTTGAACTCTCAATCTTTACGTGCCGT	8797	-
fragment_6_R	49	ACGGCACGTAAAGATTGAGAGTTCAAGTAcctaccctacgtcctcctgc	14991	23788
fragment_7_F	29	ACTCTCAATCTTTACGTGCCGTCAATCTA	8766	-
fragment_7_R	49	TAGATTGACGGCACGTAAAGATTGAGAGTcctaccctacgtcctcctgc	15022	23788
fragment_8_F	29	ATCTTTACGTGCCGTCAATCTACGCCGAT	8807	-
fragment_8_R	49	ATCGGCGTAGATTGACGGCACGTAAAGATcctaccctacgtcctcctgc	14982	23789
fragment_9_F	29	CGTGCCGTCAATCTACGCCGATTTTGTTT	8829	-
fragment_9_R	49	AAACAAAATCGGCGTAGATTGACGGCACGcctaccctacgtcctcctgc	14961	23790
fragment_10_F	29	TCAATCTACGCCGATTTTGTTTAATGTTC	8827	-
fragment_10_R	49	GAACATTAAACAAAATCGGCGTAGATTGAcctaccctacgtcctcctgc	14959	23786
fragment_11_F	29	ACGCCGATTTTGTTTAATGTTCAAGGAAC	8911	-
fragment_11_R	49	GTTCCTTGAACATTAAACAAAATCGGCGTcctaccctacgtcctcctgc	14876	23787
fragment_12_F	29	TTTTGTTTAATGTTCAAGGAACCGTCTCG	8892	-
fragment_12_R	49	CGAGACGGTTCCTTGAACATTAAACAAAAcctaccctacgtcctcctgc	14895	23787
fragment_13_F	29	TAATGTTCAAGGAACCGTCTCGTACAGTG	8921	-
fragment_13_R	49	CACTGTACGAGACGGTTCCTTGAACATTAcctaccctacgtcctcctgc	14868	23789
fragment_14_F	29	CAAGGAACCGTCTCGTACAGTGGGACACA	8925	-
fragment_14_R	49	TGTGTCCCACTGTACGAGACGGTTCCTTGcctaccctacgtcctcctgc	14867	23792

The sequences of the linker, and its complement, are shown in lower case. Data highlighted in red were used in the Supplementary Methods. F = forward strand; R = reverse strand.

Table S3. SCO3205 binding to fragments of the *sco3204-3205* intergenic region

screening oligo	replicate 1				replicate 2			
	DNA captured (RU)	protein binding			DNA captured (RU)	protein binding		
		theoretical R_{max} (RU)	measured R (RU)	% of R_{max} measured		theoretical R_{max} (RU)	measured R (RU)	% of R_{max} measured
SCO3205 protein at 10 nM								
1	384.6	517.4	0.4	0.1	427	574.5	0.1	0.0
2	408.2	549.3	0.2	0.0	435.5	586.0	0.0	0.0
3	409.1	550.5	0.4	0.1	441.1	593.6	0.0	0.0
4	410.4	552.4	36.3	6.6	442.3	595.3	36.4	6.1
5	400.0	538.4	139.5	25.9	431.5	580.8	137.7	23.7
6	382.2	514.4	140.1	27.2	403	542.4	137.3	25.3
7	413.3	556.3	2	0.4	435.6	586.3	1.4	0.2
8	386.4	520.0	0.3	0.1	411.7	554.1	0.2	0.04
9	414.4	557.7	0.2	0.0	432.6	582.2	0.1	0.0
10	407.3	548.2	3	0.5	428.2	576.4	2.7	0.5
11	422.8	569.1	138.8	24.4	455.8	613.5	135.3	22.1
12	415.7	559.5	139.4	24.9	436.9	588.0	135.6	23.1
13	416.9	561.1	0.1	0.0	439.8	591.9	0.0	0.0
14	400.7	539.2	4.6	0.9	416.7	560.7	2.5	0.5
SCO3205 protein at 50 nM								
1	413.0	555.6	1.5	0.3	431.6	580.7	1.1	0.2
2	423.2	569.4	0.9	0.2	444.1	597.6	0.7	0.1
3	421.8	567.6	1.3	0.2	442.4	595.3	0.9	0.2
4	423.4	569.9	148.8	26.1	445.5	599.6	157.3	26.2
5	413.2	556.1	637.9	114.7	433.4	583.3	666.3	114.2
6	389.0	523.6	590.5	112.8	403.4	542.9	608.0	112.0
7	427.4	575.2	10.1	1.8	440.3	592.6	8.9	1.5
8	394.8	531.3	2.7	0.5	409.3	550.9	2.0	0.4
9	425.9	573.2	1.5	0.3	439.0	590.8	1.2	0.2
10	419.7	564.9	15.0	2.7	434.2	584.4	15.2	2.6
11	432.7	582.4	626.3	107.5	454.0	611.1	650.1	106.4
12	428.1	576.2	723.3	125.5	444.1	597.7	743.6	124.4
13	427.5	575.3	1.8	0.3	440.0	592.2	1.5	0.3
14	399.2	537.2	2.4	0.4	415.9	559.7	0.9	0.2
SCO3205 protein at 100 nM								
1	420.9	566.3	3.3	0.6	434.9	585.1	2.9	0.5
2	429.3	577.6	2.6	0.5	450.5	606.2	2.3	0.4
3	431.1	580.1	3.1	0.5	450.8	606.7	2.8	0.5
4	438.4	590.0	213.1	36.1	452.8	609.4	215.4	35.3
5	420.1	565.4	655.8	116.0	438.4	590.1	682.2	115.6
6	400.7	539.3	616.0	114.2	409.1	550.6	625.5	113.6
7	431.2	580.4	19.1	3.3	444.0	597.6	17.0	2.8
8	403.9	543.6	5.6	1.0	411.8	554.2	4.9	0.9
9	428.8	577.1	3.4	0.6	444.2	597.8	3.3	0.6
10	426.4	573.9	28.9	5.0	440.3	592.6	29.6	5.0
11	448.2	603.3	664.4	110.1	457.6	615.9	672.9	109.3
12	437.2	588.5	751.2	127.7	443.5	596.9	757.1	126.8
13	435.9	586.7	4.2	0.7	446.8	601.3	3.9	0.6
14	400.7	539.2	4.6	0.9	416.7	560.7	2.5	0.5

Data highlighted in red were used in the Supplementary Methods, which explains how "theoretical R_{max}" and "% of R_{max} measured" were calculated.

Table S4. Sequences of footprinting oligomers used to define the boundaries of O_{3204}

footprinting oligo	length (bases)	sequence 5' to 3'	molecular mass (Da)	F+R annealed molecular mass (Da)
O_{3204}_RH_F	36	ACTCCAATACTTGAACTCTCAATCTTTACGTGCCGT	10909	-
O_{3204}_RH_R	56	ACGGCACGTAAAGATTGAGAGTTCAAGTATTGGAGTcctaccctacgtcctcctgc	17204	28113
O_{3204}_RH_Δ2_F	34	ACTCCAATACTTGAACTCTCAATCTTTACGTGCC	10276	-
O_{3204}_RH_Δ2_R	54	GGCACGTAAAGATTGAGAGTTCAAGTATTGGAGTcctaccctacgtcctcctgc	16602	26878
O_{3204}_RH_Δ4_F	32	ACTCCAATACTTGAACTCTCAATCTTTACGTG	9697	-
O_{3204}_RH_Δ4_R	52	CACGTAAAGATTGAGAGTTCAAGTATTGGAGTcctaccctacgtcctcctgc	15943	25640
O_{3204}_RH_Δ6_F	30	ACTCCAATACTTGAACTCTCAATCTTTACG	9064	-
O_{3204}_RH_Δ6_R	50	CGTAAAGATTGAGAGTTCAAGTATTGGAGTcctaccctacgtcctcctgc	15341	24405
O_{3204}_RH_Δ8_F	28	ACTCCAATACTTGAACTCTCAATCTTTA	8445	-
O_{3204}_RH_Δ8_R	48	TAAAGATTGAGAGTTCAAGTATTGGAGTcctaccctacgtcctcctgc	14722	23167
O_{3204}_RH_Δ10_F	26	ACTCCAATACTTGAACTCTCAATCTT	7828	-
O_{3204}_RH_Δ10_R	46	AAGATTGAGAGTTCAAGTATTGGAGTcctaccctacgtcctcctgc	14105	21933
O_{3204}_RH_Δ12_F	24	ACTCCAATACTTGAACTCTCAATC	7219	-
O_{3204}_RH_Δ12_R	44	GATTGAGAGTTCAAGTATTGGAGTcctaccctacgtcctcctgc	13478	20697
O_{3204}_RH_Δ14_F	22	ACTCCAATACTTGAACTCTCAA	6626	-
O_{3204}_RH_Δ14_R	42	TTGAGAGTTCAAGTATTGGAGTcctaccctacgtcctcctgc	12835	19461
O_{3204}_RH_Δ16_F	20	ACTCCAATACTTGAACTCTC	5999	-
O_{3204}_RH_Δ16_R	40	GAGAGTTCAAGTATTGGAGTcctaccctacgtcctcctgc	12227	18226
O_{3204}_LH_F	36	ACGGCACGTAAAGATTGAGAGTTCAAGTATTGGAGT	11217	-
O_{3204}_LH_R	56	ACTCCAATACTTGAACTCTCAATCTTTACGTGCCGTcctaccctacgtcctcctgc	16897	28114
O_{3204}_LH_Δ2_F	34	ACGGCACGTAAAGATTGAGAGTTCAAGTATTGGA	10583	-
O_{3204}_LH_Δ2_R	54	TCCAATACTTGAACTCTCAATCTTTACGTGCCGTcctaccctacgtcctcctgc	16294	26877
O_{3204}_LH_Δ4_F	32	ACGGCACGTAAAGATTGAGAGTTCAAGTATTG	9941	-
O_{3204}_LH_Δ4_R	52	CAATACTTGAACTCTCAATCTTTACGTGCCGTcctaccctacgtcctcctgc	15701	25642
O_{3204}_LH_Δ6_F	30	ACGGCACGTAAAGATTGAGAGTTCAAGTAT	9307	-
O_{3204}_LH_Δ6_R	50	ATACTTGAACTCTCAATCTTTACGTGCCGTcctaccctacgtcctcctgc	15098	24405
O_{3204}_LH_Δ8_F	28	ACGGCACGTAAAGATTGAGAGTTCAAGT	8690	-
O_{3204}_LH_Δ8_R	48	ACTTGAACTCTCAATCTTTACGTGCCGTcctaccctacgtcctcctgc	14481	23171
O_{3204}_LH_Δ10_F	26	ACGGCACGTAAAGATTGAGAGTTCAA	8056	-
O_{3204}_LH_Δ10_R	46	TTGAACTCTCAATCTTTACGTGCCGTcctaccctacgtcctcctgc	13878	21934
O_{3204}_LH_Δ12_F	24	ACGGCACGTAAAGATTGAGAGTTC	7430	-
O_{3204}_LH_Δ12_R	44	GAACTCTCAATCTTTACGTGCCGTcctaccctacgtcctcctgc	13270	20700
O_{3204}_LH_Δ14_F	22	ACGGCACGTAAAGATTGAGAGT	6836	-
O_{3204}_LH_Δ14_R	42	ACTCTCAATCTTTACGTGCCGTcctaccctacgtcctcctgc	12627	19463
O_{3204}_LH_Δ16_F	20	ACGGCACGTAAAGATTGAGA	6203	-
O_{3204}_LH_Δ16_R	40	TCTCAATCTTTACGTGCCGTcctaccctacgtcctcctgc	12024	18227

The sequence of the linker complement is shown in lower case.

Table S5. SCO3205 binding to O_{3204} footprinting oligomers

footprinting oligo	SCO3205 protein at 10 nM				SCO3205 protein at 50 nM				SCO3205 protein at 100 nM			
	DNA captured (RU)	protein binding			DNA captured (RU)	protein binding			DNA captured (RU)	protein binding		
		theoretical R_{max} (RU)	measured R (RU)	% of R_{max} measured		theoretical R_{max} (RU)	measured R (RU)	% of R_{max} measured		theoretical R_{max} (RU)	measured R (RU)	% of R_{max} measured
O_{3204}_RH	451.6	514.3	129.1	25.1	446.9	508.9	572.7	112.5	438.7	499.6	574.9	115.1
O_{3204}_RH_Δ2	439.4	523.4	127.5	24.4	436.6	520.1	556.2	106.9	427.9	509.7	559.0	109.7
O_{3204}_RH_Δ4	430.2	537.2	127.9	23.8	426.7	532.8	560.8	105.3	418.2	522.2	565.8	108.3
O_{3204}_RH_Δ6	431.7	566.3	127.4	22.5	429.8	563.8	656.8	116.5	420.3	551.4	658.4	119.4
O_{3204}_RH_Δ8	410.7	567.6	126.4	22.3	405.9	560.9	627.4	111.8	399.6	552.2	632.6	114.6
O_{3204}_RH_Δ10	408.2	595.9	122.4	20.5	403.7	589.3	664.5	112.8	397.4	580.1	672.7	116.0
O_{3204}_RH_Δ12	426.5	659.8	117.0	17.7	424.2	656.2	576.7	87.9	417.2	645.4	598.5	92.7
O_{3204}_RH_Δ14	418.5	688.5	41.5	6.0	417.8	687.3	207.6	30.2	411.2	676.5	279.4	41.3
O_{3204}_RH_Δ16	415.7	730.2	4.4	0.6	414.0	727.2	30.0	4.1	408.5	717.6	56.3	7.8
O_{3204}_LH	435.1	495.5	122.3	24.7	431.2	491.1	523.1	106.5	426.2	485.4	535.7	110.4
O_{3204}_LH_Δ2	419.2	499.4	121.7	24.4	416.2	495.8	552.9	111.5	409.4	487.7	563.9	115.6
O_{3204}_LH_Δ4	428.7	535.3	120.2	22.5	426.7	532.8	572.9	107.5	420.7	525.3	587.6	111.9
O_{3204}_LH_Δ6	425.2	557.8	105.2	18.9	424.0	556.2	466.6	83.9	417.7	548.0	481.7	87.9
O_{3204}_LH_Δ8	414.6	572.9	88.5	15.4	412.6	570.1	435.1	76.3	406.0	561.0	451.2	80.4
O_{3204}_LH_Δ10	425.2	620.6	1.0	0.2	424.9	620.2	15.0	2.4	418.5	610.9	29.0	4.7
O_{3204}_LH_Δ12	415.3	642.3	-0.2	0.0	414.0	640.3	5.1	0.8	407.8	630.7	9.4	1.5
O_{3204}_LH_Δ14	410.8	675.8	-0.3	0.0	412.4	678.4	4.2	0.6	406.0	667.9	9.4	1.4
O_{3204}_LH_Δ16	424.9	746.3	-0.1	0.0	426.6	749.3	4.7	0.6	419.1	736.2	10.1	1.4

An explanation of how "theoretical R_{max}" and "% of R_{max} measured" were calculated is given in the Supplementary Methods.

Table S6. Sequences of footprinting oligomers used to define the boundaries of O_{3205}

footprinting oligo	length (bases)	sequence 5' to 3'	molecular mass (Da)	F+R annealed molecular mass (Da)
O_{3205}_RH_F	36	ACGCCGATTTTGTTTAATGTTCAAGGAACCGTCTCG	11045	-
O_{3205}_RH_R	56	CGAGACGGTTCCTTGAACATTAAACAAAATCGGCGTcctaccctacgtcctcctgc	17069	28114
O_{3205}_RH_Δ2_F	34	ACGCCGATTTTGTTTAATGTTCAAGGAACCGTCT	10427	-
O_{3205}_RH_Δ2_R	54	AGACGGTTCCTTGAACATTAAACAAAATCGGCGTcctaccctacgtcctcctgc	16451	26878
O_{3205}_RH_Δ4_F	32	ACGCCGATTTTGTTTAATGTTCAAGGAACCGT	9833	-
O_{3205}_RH_Δ4_R	52	ACGGTTCCTTGAACATTAAACAAAATCGGCGTcctaccctacgtcctcctgc	15808	25641
O_{3205}_RH_Δ6_F	30	ACGCCGATTTTGTTTAATGTTCAAGGAACC	9200	-
O_{3205}_RH_Δ6_R	50	GGTTCCTTGAACATTAAACAAAATCGGCGTcctaccctacgtcctcctgc	15206	24406
O_{3205}_RH_Δ8_F	28	ACGCCGATTTTGTTTAATGTTCAAGGAA	8621	-
O_{3205}_RH_Δ8_R	48	TTCCTTGAACATTAAACAAAATCGGCGTcctaccctacgtcctcctgc	14547	23168
O_{3205}_RH_Δ10_F	26	ACGCCGATTTTGTTTAATGTTCAAGG	7995	-
O_{3205}_RH_Δ10_R	46	CCTTGAACATTAAACAAAATCGGCGTcctaccctacgtcctcctgc	13939	21934
O_{3205}_RH_Δ12_F	24	ACGCCGATTTTGTTTAATGTTCAA	7336	-
O_{3205}_RH_Δ12_R	44	TTGAACATTAAACAAAATCGGCGTcctaccctacgtcctcctgc	13360	20696
O_{3205}_RH_Δ14_F	22	ACGCCGATTTTGTTTAATGTTC	6709	-
O_{3205}_RH_Δ14_R	42	GAACATTAAACAAAATCGGCGTcctaccctacgtcctcctgc	12752	19461
O_{3205}_LH_F	36	CGAGACGGTTCCTTGAACATTAAACAAAATCGGCGT	11082	-
O_{3205}_LH_R	56	ACGCCGATTTTGTTTAATGTTCAAGGAACCGTCTCGcctaccctacgtcctcctgc	17033	28115
O_{3205}_LH_Δ2_F	34	CGAGACGGTTCCTTGAACATTAAACAAAATCGGC	10448	-
O_{3205}_LH_Δ2_R	54	GCCGATTTTGTTTAATGTTCAAGGAACCGTCTCGcctaccctacgtcctcctgc	16430	26878
O_{3205}_LH_Δ4_F	32	CGAGACGGTTCCTTGAACATTAAACAAAATCG	9830	-
O_{3205}_LH_Δ4_R	52	CGATTTTGTTTAATGTTCAAGGAACCGTCTCGcctaccctacgtcctcctgc	15812	25642
O_{3205}_LH_Δ6_F	30	CGAGACGGTTCCTTGAACATTAAACAAAAT	9211	-
O_{3205}_LH_Δ6_R	50	ATTTTGTTTAATGTTCAAGGAACCGTCTCGcctaccctacgtcctcctgc	15193	24404
O_{3205}_LH_Δ8_F	28	CGAGACGGTTCCTTGAACATTAAACAAA	8594	-
O_{3205}_LH_Δ8_R	48	TTTGTTTAATGTTCAAGGAACCGTCTCGcctaccctacgtcctcctgc	14576	23170
O_{3205}_LH_Δ10_F	26	CGAGACGGTTCCTTGAACATTAAACA	7967	-
O_{3205}_LH_Δ10_R	46	TGTTTAATGTTCAAGGAACCGTCTCGcctaccctacgtcctcctgc	13967	21934
O_{3205}_LH_Δ12_F	24	CGAGACGGTTCCTTGAACATTAAA	7365	-
O_{3205}_LH_Δ12_R	44	TTTAATGTTCAAGGAACCGTCTCGcctaccctacgtcctcctgc	13334	20699
O_{3205}_LH_Δ14_F	22	CGAGACGGTTCCTTGAACATTA	6738	-
O_{3205}_LH_Δ14_R	42	TAATGTTCAAGGAACCGTCTCGcctaccctacgtcctcctgc	12725	19463

The sequence of the linker complement is shown in lower case.

Table S7. SCO3205 binding to O_{3205} footprinting oligomers

oligo	SCO3205 protein at 10 nM				SCO3205 protein at 50 nM				SCO3205 protein at 100 nM			
	DNA captured (RU)	protein binding			DNA captured (RU)	protein binding			DNA captured (RU)	protein binding		
		theoretical R_{max} (RU)	measured R (RU)	% of R_{max} measured		theoretical R_{max} (RU)	measured R (RU)	% of R_{max} measured		theoretical R_{max} (RU)	measured R (RU)	% of R_{max} measured
O_{3205}_RH*	475.6	541.6	109.3	20.2	476.2	542.3	446.7	82.4	469.9	535.1	453.8	84.8
O_{3205}_RH_Δ2	436.1	519.5	108.7	20.9	435.3	518.5	599.7	115.7	427.9	509.7	602.1	118.1
O_{3205}_RH_Δ4	419.6	523.9	108.1	20.6	417.6	521.4	560.8	107.6	412.7	515.3	565.7	109.8
O_{3205}_RH_Δ6	405.2	531.5	107.3	20.2	406.3	533.0	609.5	114.4	401.0	526.0	614.2	116.8
O_{3205}_RH_Δ8	408.3	564.2	98.0	17.4	406.5	561.7	506.5	90.2	403.3	557.3	523.8	94.0
O_{3205}_RH_Δ10	414.9	605.6	83.0	13.7	412.8	602.5	454.2	75.4	409.9	598.3	484.8	81.0
O_{3205}_RH_Δ12	414.4	641.1	8.3	1.3	414.3	640.9	117.9	18.4	410.6	635.2	190.2	29.9
O_{3205}_RH_Δ14	417.3	686.5	0.9	0.1	416.3	684.9	17.2	2.5	413.6	680.4	34.5	5.1
O_{3205}_LH	459.9	523.7	102.6	19.6	458.7	522.3	548.7	105.0	455.0	518.1	558.2	107.7
O_{3205}_LH_Δ2	443.9	528.8	101.1	19.1	442.2	526.7	577.7	109.7	438.1	521.9	586.3	112.4
O_{3205}_LH_Δ4	446.9	558.0	132.5	23.7	444.0	554.4	587.7	106.0	441.3	551.0	595.9	108.1
O_{3205}_LH_Δ6	439.0	575.9	132.2	23.0	437.0	573.3	565.8	98.7	432.6	567.5	576.1	101.5
O_{3205}_LH_Δ8	450.0	621.8	29.7	4.8	450.4	622.4	389.7	62.6	451.9	624.4	427.4	68.4
O_{3205}_LH_Δ10	417.2	609.0	92.0	15.1	415.1	605.9	381.5	63.0	411.2	600.2	411.8	68.6
O_{3205}_LH_Δ12	426.3	659.4	14.8	2.2	424.5	656.6	94.3	14.4	420.5	650.4	143.4	22.0
O_{3205}_LH_Δ14	426.0	700.8	0.1	0.0	424.7	698.6	4.9	0.7	420.0	690.9	10.9	1.6

An explanation of how "theoretical R_{max}" and "% of R_{max} measured" were calculated is given in the Supplementary Methods. *O_{3205}_RH gave an anomalously low normalized maximum response, which we are unable to explain.

Table S8. Sequences of oligomers used for affinity measurements of O_{3204} and O_{3205}

affinity oligo	length (bases)	sequence 5' to 3'	molecular mass (Da)	F+R annealed molecular mass (Da)
O_{3204}_24mer_F	24	CAATACTTGAACTCTCAATCTTTA	7249	-
O_{3204}_24mer_R	44	TAAAGATTGAGAGTTCAAGTATTGcctaccctacgtcctcctgc	13446	20695
O_{3205}_24mer_F	24	ATTTTGTTTAATGTTCAAGGAACC	7357	-
O_{3205}_24mer_R	44	GGTTCCTTGAACATTAAACAAAATcctaccctacgtcctcctgc	13331	20687

The sequence of the linker complement is shown in lower case.

Table S9. Sequences of oligomers used for testing putative members of the SCO3205 regulon

operator oligo	length (bases)	sequence 5' to 3'	molecular mass (Da)	F+R annealed molecular mass (Da)
O_{5049}_24mer_F	24	AAGAAGTTCAAGCTTCAACCAAAA	7351	-
O_{5049}_24mer_R	44	TTTTGGTTGAAGCTTGAACTTCTTcctaccctacgtcctcctgc	13345	20696
O_{5533}_24mer_F	24	TTGATGTTGAATGTTCAACCAGTC	7361	-
O_{5533}_24mer_R	44	GACTGGTTGAACATTCAACATCAAcctaccctacgtcctcctgc	13336	20697
O_{7815}_24mer_F	24	GACCTGTTCAAAGCTCAAGCAGAA	7359	-
O_{7815}_24mer_R	44	TTCTGCTTGAGCTTTGAACAGGTCcctaccctacgtcctcctgc	13340	20699

The sequence of the linker complement is shown in lower case.

Table S10. Comparison of SCO3205 binding to O_{3204} with binding to other putative regulon members

operator	SCO3205 protein at 10 nM				SCO3205 protein at 50 nM				SCO3205 protein at 100 nM			
	DNA captured (RU)	protein binding			DNA captured (RU)	protein binding			DNA captured (RU)	protein binding		
		theoretical R_{max} (RU)	measured R (RU)	% of R_{max} measured		theoretical R_{max} (RU)	measured R (RU)	% of R_{max} measured		theoretical R_{max} (RU)	measured R (RU)	% of R_{max} measured
O_{3204}_24mer	403.7	667.1	189.7	28.4	426.7	660.1	664.7	100.7	431.5	667.6	674.3	101.0
O_{5049}_24mer	396.5	624.5	170.1	27.2	404.1	625.1	573.5	91.7	404.4	625.6	595.5	95.2
O_{5533}_24mer	408.0	613.3	191.2	31.2	396.8	613.8	717.3	116.9	397	614.1	725.4	118.1
O_{7815}_24mer	431.2	631.1	80.3	12.7	408.6	632.0	244.4	38.7	408.3	631.5	306	48.5

An explanation of how "theoretical R_{max}" and "% of R_{max} measured" were calculated is given in the Supplementary Methods.

Table S11. Comparison of SCO3205 dissociation from O_{3204} with dissociation from other putative regulon members

operator	SCO3205 protein at 10 nM			SCO3205 protein at 50 nM			SCO3205 protein at 100 nM		
	% remaining after dissociation	% remaining after wash 1	% remaining after wash 2	% remaining after dissociation	% remaining after wash 1	% remaining after wash 2	% remaining after dissociation	% remaining after wash 1	% remaining after wash 2
O_{3204}_24mer	97.9	83.9	78.1	82.2	67.2	60.8	81.4	66.4	60.1
O_{5049}_24mer	80.5	0.6	0.0	53.1	1.5	0.2	51.6	1.6	0.3
O_{5533}_24mer	97.0	78.2	71.1	87.2	73.1	65.5	86.7	72.6	64.9
O_{7815}_24mer	0.0	0.0	0.0	1.0	0.0	0.0	1.3	0.0	0.0

Table S12. Sequences of oligomers used for testing substitutions within the consensus motif of O_{3204}

consensus testing oligo	length (bases)	sequence 5' to 3'	molecular mass (Da)	F+R annealed molecular mass (Da)
O_{3204}_WT_F	24	CAATACTTGAACTCTCAATCTTTA	7249	-
O_{3204}_WT_R	44	TAAAGATTGAGAGTTCAAGTATTGcctaccctacgtcctcctgc	13446	20695
O_{3204}_T6G_F	24	CAATACGTGAACTCTCAATCTTTA	7275	-
O_{3204}_T6G_R	44	TAAAGATTGAGAGTTCACGTATTGcctaccctacgtcctcctgc	13422	20697
O_{3204}_T6C_F	24	CAATACCTGAACTCTCAATCTTTA	7234	-
O_{3204}_T6C_R	44	TAAAGATTGAGAGTTCAGGTATTGcctaccctacgtcctcctgc	13462	20696
O_{3204}_T6A_F	24	CAATACATGAACTCTCAATCTTTA	7259	-
O_{3204}_T6A_R	44	TAAAGATTGAGAGTTCATGTATTGcctaccctacgtcctcctgc	13437	20696
O_{3204}_T7G_F	24	CAATACTGGAACTCTCAATCTTTA	7275	-
O_{3204}_T7G_R	44	TAAAGATTGAGAGTTCCAGTATTGcctaccctacgtcctcctgc	13422	20697
O_{3204}_T7C_F	24	CAATACTCGAACTCTCAATCTTTA	7234	-
O_{3204}_T7C_R	44	TAAAGATTGAGAGTTCGAGTATTGcctaccctacgtcctcctgc	13462	20696
O_{3204}_T7A_F	24	CAATACTAGAACTCTCAATCTTTA	7259	-
O_{3204}_T7A_R	44	TAAAGATTGAGAGTTCTAGTATTGcctaccctacgtcctcctgc	13437	20696
O_{3204}_A9T_F	24	CAATACTTGTACTCTCAATCTTTA	7240	-
O_{3204}_A9T_R	44	TAAAGATTGAGAGTACAAGTATTGcctaccctacgtcctcctgc	13455	20695
O_{3204}_A9G_F	24	CAATACTTGGACTCTCAATCTTTA	7265	-
O_{3204}_A9G_R	44	TAAAGATTGAGAGTCCAAGTATTGcctaccctacgtcctcctgc	13431	20696
O_{3204}_A9C_F	24	CAATACTTGCACTCTCAATCTTTA	7225	-
O_{3204}_A9C_R	44	TAAAGATTGAGAGTGCAAGTATTGcctaccctacgtcctcctgc	13471	20696
O_{3204}_A10T_F	24	CAATACTTGATCTCTCAATCTTTA	7240	-
O_{3204}_A10T_R	44	TAAAGATTGAGAGATCAAGTATTGcctaccctacgtcctcctgc	13455	20695
O_{3204}_A10G_F	24	CAATACTTGAGCTCTCAATCTTTA	7265	-
O_{3204}_A10G_R	44	TAAAGATTGAGAGCTCAAGTATTGcctaccctacgtcctcctgc	13431	20696
O_{3204}_A10C_F	24	CAATACTTGACCTCTCAATCTTTA	7225	-
O_{3204}_A10C_R	44	TAAAGATTGAGAGGTCAAGTATTGcctaccctacgtcctcctgc	13471	20696
O_{3204}_T14G_F	24	CAATACTTGAACTCGCAATCTTTA	7275	-
O_{3204}_T14G_R	44	TAAAGATTGCGAGTTCAAGTATTGcctaccctacgtcctcctgc	13422	20697
O_{3204}_T14C_F	24	CAATACTTGAACTCCCAATCTTTA	7234	-
O_{3204}_T14C_R	44	TAAAGATTGGGAGTTCAAGTATTGcctaccctacgtcctcctgc	13462	20696
O_{3204}_T14A_F	24	CAATACTTGAACTCACAATCTTTA	7259	-
O_{3204}_T14A_R	44	TAAAGATTGTGAGTTCAAGTATTGcctaccctacgtcctcctgc	13437	20696
O_{3204}_C15T_F	24	CAATACTTGAACTCTTAATCTTTA	7265	-
O_{3204}_C15T_R	44	TAAAGATTAAGAGTTCAAGTATTGcctaccctacgtcctcctgc	13430	20695
O_{3204}_C15G_F	24	CAATACTTGAACTCTGAATCTTTA	7290	-
O_{3204}_C15G_R	44	TAAAGATTCAGAGTTCAAGTATTGcctaccctacgtcctcctgc	13406	20696
O_{3204}_C15A_F	24	CAATACTTGAACTCTAAATCTTTA	7274	-
O_{3204}_C15A_R	44	TAAAGATTTAGAGTTCAAGTATTGcctaccctacgtcctcctgc	13421	20695
O_{3204}_A16T_F	24	CAATACTTGAACTCTCTATCTTTA	7249	-
O_{3204}_A16T_R	44	TAAAGATAGAGAGTTCAAGTATTGcctaccctacgtcctcctgc	13446	20695
O_{3204}_A16G_F	24	CAATACTTGAACTCTCGATCTTTA	7265	-
O_{3204}_A16G_R	44	TAAAGATCGAGAGTTCAAGTATTGcctaccctacgtcctcctgc	13431	20696
O_{3204}_A16C_F	24	CAATACTTGAACTCTCCATCTTTA	7225	-
O_{3204}_A16C_R	44	TAAAGATGGAGAGTTCAAGTATTGcctaccctacgtcctcctgc	13471	20696
O_{3204}_A17T_F	24	CAATACTTGAACTCTCATTCTTTA	7240	-
O_{3204}_A17T_R	44	TAAAGAATGAGAGTTCAAGTATTGcctaccctacgtcctcctgc	13455	20695
O_{3204}_A17G_F	24	CAATACTTGAACTCTCAGTCTTTA	7265	-
O_{3204}_A17G_R	44	TAAAGACTGAGAGTTCAAGTATTGcctaccctacgtcctcctgc	13431	20696
O_{3204}_A17C_F	24	CAATACTTGAACTCTCACTCTTTA	7225	-
O_{3204}_A17C_R	44	TAAAGAGTGAGAGTTCAAGTATTGcctaccctacgtcctcctgc	13471	20696

The sequence of the linker complement is shown in lower case. The sequence numbering is relative to the 22-mer used to determine the crystal structure (Figure 5).

Table S13. The effects of substitutions within the consensus motif of O_{3204} on the binding of SCO3205

SCO3205 protein at 50 nM							
consensus testing oligo	DNA captured (RU)	protein binding			protein dissociation		
		theoretical R_{max} (RU)	measured R (RU)	% of R_{max} measured	% remaining after dissociation	% remaining after wash 1	% remaining after wash 2
O_{3204}_WT	432.5	669.1	663.3	99.1	82.4	67.3	60.9
O_{3204}_T6G	422.5	653.6	634.3	97.1	70.8	20.1	7.2
O_{3204}_T6C	431.0	666.7	604.8	90.7	60.8	8.5	1.8
O_{3204}_T6A	417.1	645.2	575.6	89.2	63.6	10.1	1.9
O_{3204}_WT	432.5	669.1	661.1	98.8	82.4	66.8	60.4
O_{3204}_T7G	426.2	659.3	580.9	88.1	62.3	8.5	1.8
O_{3204}_T7C	426.7	660.1	490.1	74.2	23.0	-0.1	-0.7
O_{3204}_T7A	415.0	642.0	619.4	96.5	67.5	15.3	4.0
O_{3204}_A9T	419.3	648.7	645.1	99.4	71.3	16.9	5.1
O_{3204}_A9G	438.7	678.7	534.3	78.7	31.0	-0.9	-1.3
O_{3204}_A9C	439.1	679.3	631.8	93.0	66.5	6.6	0.8
O_{3204}_WT	433.9	671.3	663.8	98.9	82.1	66.2	60.3
O_{3204}_A10T	424.9	657.3	685.2	104.2	76.9	48.6	34.5
O_{3204}_A10G	412.8	638.6	610.8	95.6	70.5	31.1	15.9
O_{3204}_A10C	417.0	645.1	619.4	96.0	67.3	13.5	3.6
O_{3204}_WT	431.8	668.0	653.8	97.9	82.3	66.5	60.0
O_{3204}_WT	431.6	667.7	658.5	98.6	82.3	66.5	60.1
O_{3204}_T14G	424.1	656.0	714.9	109.0	74.5	26.2	11.2
O_{3204}_T14C	416.6	644.5	550.0	85.3	35.0	-0.5	-1.1
O_{3204}_T14A	411.0	635.8	691.4	108.7	70.7	18.8	6.6
O_{3204}_C15T	417.0	645.1	655.2	101.6	71.8	31.0	15.6
O_{3204}_C15G	418.5	647.4	617.6	95.4	55.7	4.0	0.7
O_{3204}_WT	432.6	669.3	660.2	98.6	82.4	67.3	60.9
O_{3204}_C15A	408.3	631.7	700.1	110.8	81.7	66.3	57.1
O_{3204}_A16T	431.4	667.4	693.8	104.0	72.1	24.4	10.3
O_{3204}_A16G	415.7	643.1	471.4	73.3	28.2	0.2	-0.5
O_{3204}_A16C	410.9	635.7	646.8	101.8	68.8	16.7	5.7
O_{3204}_WT	432.8	669.6	659.1	98.4	82.0	67.0	60.7
O_{3204}_A17T	433.5	670.6	672.4	100.3	74.1	35.1	19.1
O_{3204}_A17G	418.6	647.6	677.9	104.7	72.7	26.3	11.4
O_{3204}_A17C	413.9	640.3	660.5	103.2	72.1	25.8	11.3
O_{3204}_WT	432.6	669.3	658.2	98.3	82.1	67.1	60.7

An explanation of how "theoretical R_{max}" and "% of R_{max} measured" were calculated is given in the Supplementary Methods. The sequence numbering is relative to the 22-mer used to determine the crystal structure (see Figure 5).

Table S14. X-ray data collection and refinement statistics

Data set	SCO3205-DNA (22-mer)
Data collection	
Beamline	I24, Diamond Light Source, UK
Wavelength (Å)	0.9780
Detector	Pilatus 6M
Resolution range[a] (Å)	69.68 - 2.80 (2.87 - 2.80)
Space Group	$P6_5$
Cell parameters (Å)	$a = b = 70.80$, $c = 557.48$
Total no. of measured intensities[a]	324262 (6687)
Unique reflections[a]	37192 (2206)
Multiplicity[a]	8.7 (3.0)
Mean $I/\sigma(I)$[a]	13.7 (1.9)
Completeness[a] (%)	96.0 (78.2)
R_{merge}[a,b]	0.102 (0.370)
R_{meas}[a,c]	0.119 (0.488)
$CC_{½}$[a,d]	0.997 (0.608)
Wilson B value ($Å^2$)	80.9
Refinement	
Resolution range[a] (Å)	59.88 - 2.80 (2.87 - 2.80)
Reflections: working/free[e]	35209/1848
R_{work}/ R_{free}[a,f]	0.176/0.196 (0.300/0.401)
Ramachandran plot: favoured/allowed/disallowed (outliers)[g] (%)	96.6/3.1/0.3 (2)
R.m.s. bond distance deviation (Å)	0.008
R.m.s. bond angle deviation (°)	1.35
No. of protein residues (ranges): chains A/B/E/F	159 (5-163)/ 159 (5-163)/ 160 (2-154,157-163)/ 154 (7-154,158-163)
No. of DNA bases (ranges): chains C/D/G/H	22 (1-22) for all chains
No. of water molecules/phosphate ions	14/4
Mean B factors: protein/DNA/water/phosphate/overall ($Å^2$)	62.1/57.4/48.8/87.6/60.9
Refined twin fraction (h, k, l / k, h, -l)	0.67/0.33
PDB accession code	3ZPL

[a] Figures in parentheses indicate values for the outer resolution shell.

[b] $R_{merge} = \sum_{hkl} \sum_i |I_i(hkl) - \langle I(hkl)\rangle| / \sum_{hkl} \sum_i I_i(hkl)$.

[c] $R_{meas} = \sum_{hkl} [N/(N-1)]^{1/2} \times \sum_i |I_i(hkl) - \langle I(hkl)\rangle| / \sum_{hkl} \sum_i I_i(hkl)$, where $I_i(hkl)$ is the ith observation of reflection hkl, $\langle I(hkl)\rangle$ is the weighted average intensity for all observations i of reflection hkl and N is the number of observations of reflection hkl.

[d] $CC_{½}$ is the correlation coefficient between intensities taken from random halves of the dataset.

[e] The data set was split into "working" and "free" sets consisting of 95 and 5% of the data, respectively. The free set was not used for refinement.

[f] The R-factors R_{work} and R_{free} are calculated as follows: $R = \sum(| F_{obs} - F_{calc} |)/\sum| F_{obs} |$ x 100, where F_{obs} and F_{calc} are the observed and calculated structure factor amplitudes, respectively.

[g] As calculated using MolProbity (53).

Table S15. Selected structural homologs of SCO3205

Protein	Source	DNA bound	PDB code	Resolution (Å)	DALI output			R.m.s. deviation (Å)/ aligned residues		Reference
					Rank	Z-score	Identity (%)	subunit	dimer	
SCO5405	*Streptomyces coelicolor*	No	3ZMD	1.95	1	19.5	43	0.99/142	1.09/282	Stevenson *et al*., unpublished
SCO5413	*Streptomyces coelicolor*	No	4B8X	1.25	2	16.7	20	2.01/135	2.22/265	(45)
BldR	*Sulfolobus solfataricus*	No	3F3X	1.90	3	16.4	19	2.06/133	2.90/270	(55)
OhrR	*Bacillus subtilis*	Yes	1Z9C	2.64	4	16.3	15	1.97/121	2.18/233	(1)
SlyA	*Salmonella enterica*	Yes	3Q5F	2.96	5	16.1	15	1.70/138	2.66/266	(5)
MosR	*Mycobacterium tuberculosis*	Yes	4FX4	3.10	12	15.4	20	2.11/122	2.26/247	(18)

```
     KpnI NdeI
     GGTACCATATGCATCATCATCATCACCACGAAAACCTGTATTTTCAGGGCATGAACGATG
1    ---------+---------+---------+---------+---------+---------+
             M  H  H  H  H  H  H  E  N  L  Y  F  Q  G  M  N  D  E

                    SacII       PvuII
     AACCGCGTTGGCTGACCGCGGAAGAACAGCTGGTGTGGCGTAGCTATATTGAAGCGGCGA
61   ---------+---------+---------+---------+---------+---------+
      P  R  W  L  T  A  E  E  Q  L  V  W  R  S  Y  I  E  A  A  T

                                PvuII
                                   PstI             SphI
     CCCTGCTGGAAGATCATCTGGATCGTCAGCTGCAGCGTGATGCGGGCATGCCGCATGTGT
121  ---------+---------+---------+---------+---------+---------+
      L  L  E  D  H  L  D  R  Q  L  Q  R  D  A  G  M  P  H  V  Y

     ATTATGGCCTGCTGGTGAAACTGGCCGAAAGCCCGCGTCGTCGTCTGCGTATGACCGAAC
181  ---------+---------+---------+---------+---------+---------+
      Y  G  L  L  V  K  L  A  E  S  P  R  R  R  L  R  M  T  E  L

                                           PflMI
     TGGCCAAATATGCGAAAATTACCCGTAGCCGTCTGAGCCATGCGGTGGCGCGTCTGGAAA
241  ---------+---------+---------+---------+---------+---------+
      A  K  Y  A  K  I  T  R  S  R  L  S  H  A  V  A  R  L  E  K

     AAAACGGCTGGGTGCGTCGTGAAGATTGCCCGAGCGATAAACGTGGCCAGTTTGCGATTC
301  ---------+---------+---------+---------+---------+---------+
      N  G  W  V  R  R  E  D  C  P  S  D  K  R  G  Q  F  A  I  L

     TGACCGATGAAGGCTATGAAGTGCTGCGTCGTACCGCACCGGGTCATGTGGATGCGGTGC
361  ---------+---------+---------+---------+---------+---------+
      T  D  E  G  Y  E  V  L  R  R  T  A  P  G  H  V  D  A  V  R

     GTCAGGCGGTGTTTGATCGTCTGACCCCGGAACAGCAGAAAAGCCTGGGCGAAATTATGC
421  ---------+---------+---------+---------+---------+---------+
      Q  A  V  F  D  R  L  T  P  E  Q  Q  K  S  L  G  E  I  M  R

                  StuI
                      PstI
     GTATTGTGGCGGAAGGCCTGCAGCCGAGCGAAGCGGGTGCGGATCTGCCGTGGCTGCGTT
481  ---------+---------+---------+---------+---------+---------+
      I  V  A  E  G  L  Q  P  S  E  A  G  A  D  L  P  W  L  R  *

          HindIII
     AATAAAAGCTT
541  ---------+-
      *
```

Figure S1. Nucleotide sequence of the synthetic *sco3205* gene with optimized codon usage for expression in *E. coli*. The sequence contains two stop codons to ensure efficient termination. This construct was subcloned into pET21a before transformation into *E. coli* BL21 (DE3) cells. The translated amino acid sequence is shown below, where the full wild-type sequence is shown in bold and the N-terminal His-tag is shown in plain text. Also shown are potential restriction sites.

```
29bp oligos with 22bp overlap:
          1         2         3         4         5         6         7         8         9        10        11        12
0123456789012345678901234567890123456789012345678901234567890123456789012345678901234567890123456789012345678901234567890

+GCGCTCCCGGACGGCGGCGGCCATGGCTA:1
+       CGGACGGCGGCGGCCATGGCTACTCCAAT:2
+              CGGCGGCCATGGCTACTCCAATACTTGAA:3
+                     CATGGCTACTCCAATACTTGAACTCTCAA:4
+                            ACTCCAATACTTGAACTCTCAATCTTTAC:5
+                                   TACTTGAACTCTCAATCTTTACGTGCCGT:6
+                                          ACTCTCAATCTTTACGTGCCGTCAATCTA:7
+                                                 ATCTTTACGTGCCGTCAATCTACGCCGAT:8
+                                                        CGTGCCGTCAATCTACGCCGATTTTGTTT:9
+                                                               TCAATCTACGCCGATTTTGTTTAATGTTC:10
+                                                                      ACGCCGATTTTGTTTAATGTTCAAGGAAC:11
+                                                                             TTTTGTTTAATGTTCAAGGAACCGTCTCG:12
+                                                                                    TAATGTTCAAGGAACCGTCTCGTACAGTG:13
+                                                                                           CAAGGAACCGTCTCGTACAGTGGGACACA:14
+
```

Figure S2. The 119 nt sequence of the *sco3204-sco3205* intergenic region was fragmented using a Perl script, termed POOP (Perl Overlapping Oligo Producer). Part of the program output is shown above. Each fragment oligomer is 29 nt long and overlaps with its neighbour(s) by 22 nt. The 3' adenine of fragment 14 actually corresponds to the first nt of the start codon for *sco3205*. POOP also produces a text file containing all the required oligomer fragments in a format suitable for ordering for synthesis, including the complement to the ReDCaT linker (see Supplementary Table S2), which was attached to the 3’ ends of the reverse strands in all cases. POOP is available as part of this submission (Supplementary Program 1) and should run on any Unix operating system.

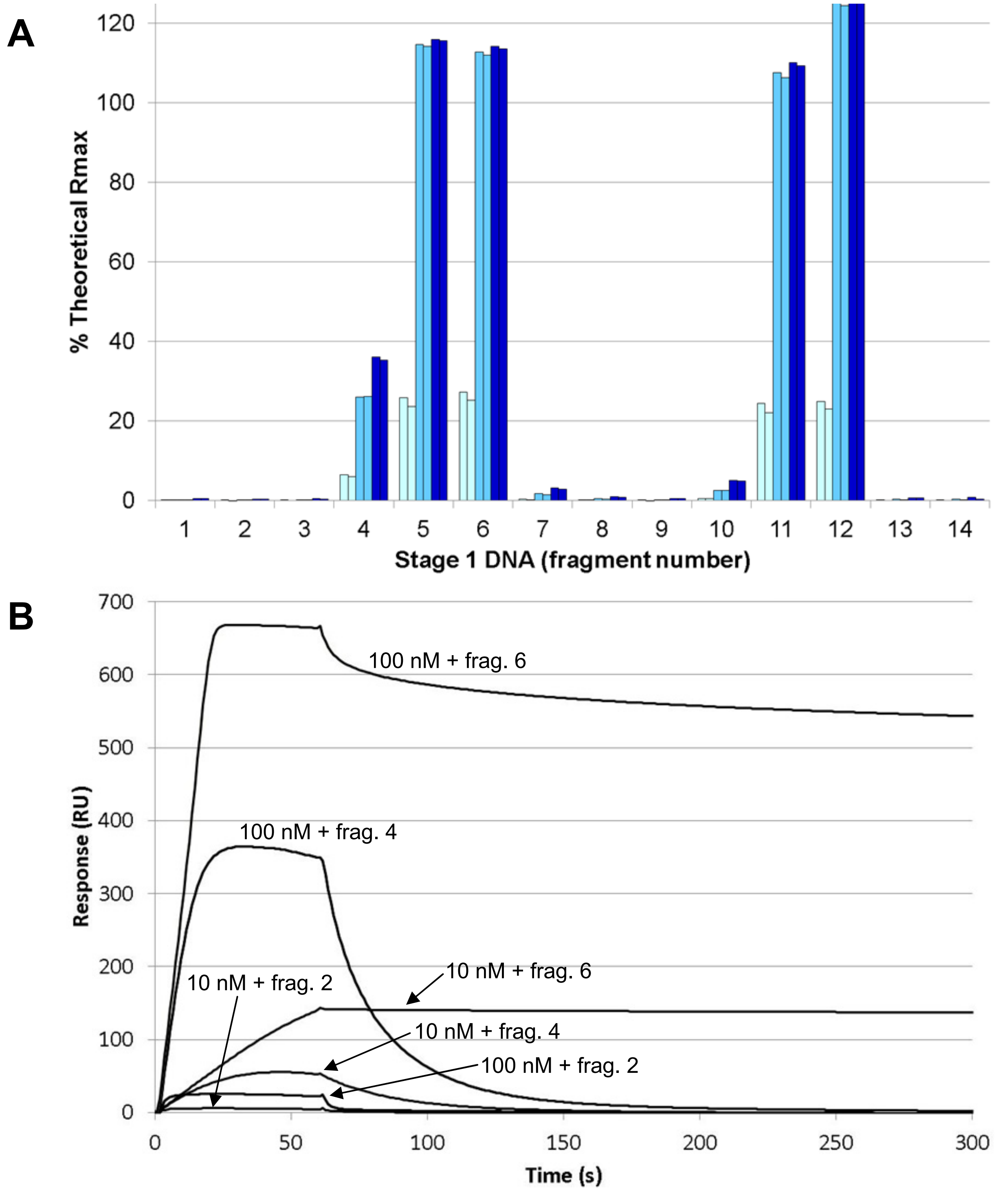

Figure S3. SPR screening for the binding of SCO3205 to fragments of the *sco3204-sco3205* intergenic region. (**A**) The responses were normalized and expressed as a percentage of the theoretical R_{max} (see Supplementary Methods). In each case, the test DNA oligomers were 29 bp in length. SCO3205 binding was measured at concentrations of 10 nM (pale blue), 50 nM (mid blue) and 100 nM (dark blue). All measurements were carried out in duplicate. The oligomer sequences and the SPR data are given in Supplementary Tables S2 and S3. (**B**) Selected sensorgrams showing protein binding and dissociation phases for a non-binding sequence (fragment 2) a partial hit (fragment 4) and a full hit (fragment 6), each at both 10 nM and 100 nM SCO3205 concentration. Note that the relatively short injection time used in the screening prevented the 10 nM injection of SCO3205 over fragment 6 from saturating.

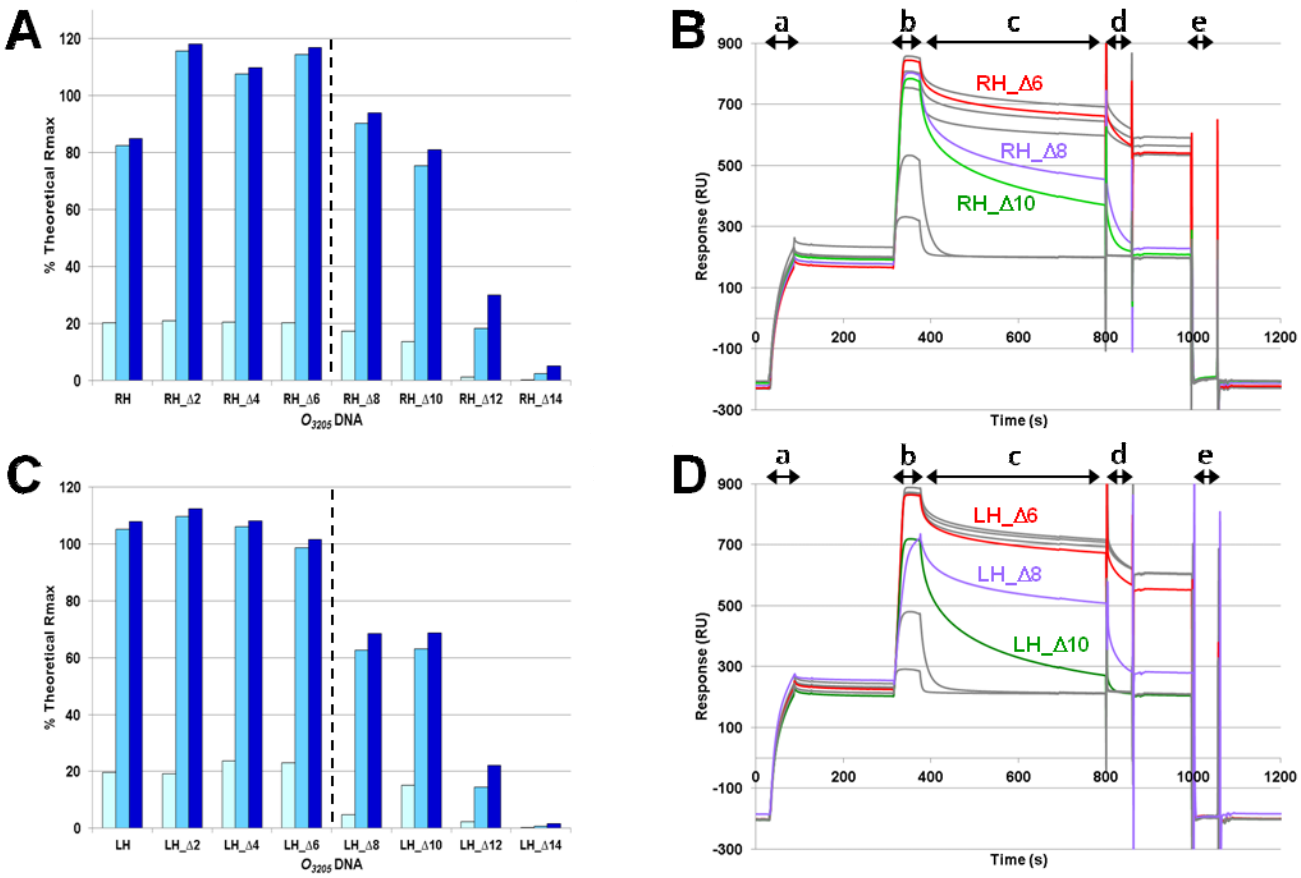

Figure S4. SPR footprinting of O_{3205}. Serially truncated oligomers were used to define the extent of the O_{3205} binding site. Panels (**A**) and (**B**) display the data for defining the right-hand border, and panels (**C**) and (**D**) display the data for defining the left-hand border. Panels (**A**) and (**C**) show the normalized responses for each test oligomer at SCO3205 concentrations of 10 nM (pale blue), 50 nM (mid blue) and 100 nM (dark blue). The vertical dashed lines indicate the proposed footprint boundaries. Panels (**B**) and (**D**) show the corresponding sensorgrams for 100 nM SCO3205 only. The complete "ReDCaT cycle" is shown for each, where: a = test DNA capture, b = protein binding, c = protein dissociation, d = salt wash, e = stripping of test DNA. Key sensorgrams are coloured, where red represents the one chosen to represent the footprint boundary, and purple and green represent the next two truncations, respectively. The remaining sensorgrams are coloured grey. N.B. No correction has been made to account for the length or quantity of test DNA captured. Thus, the maximum responses in the sensorgrams are not directly comparable.

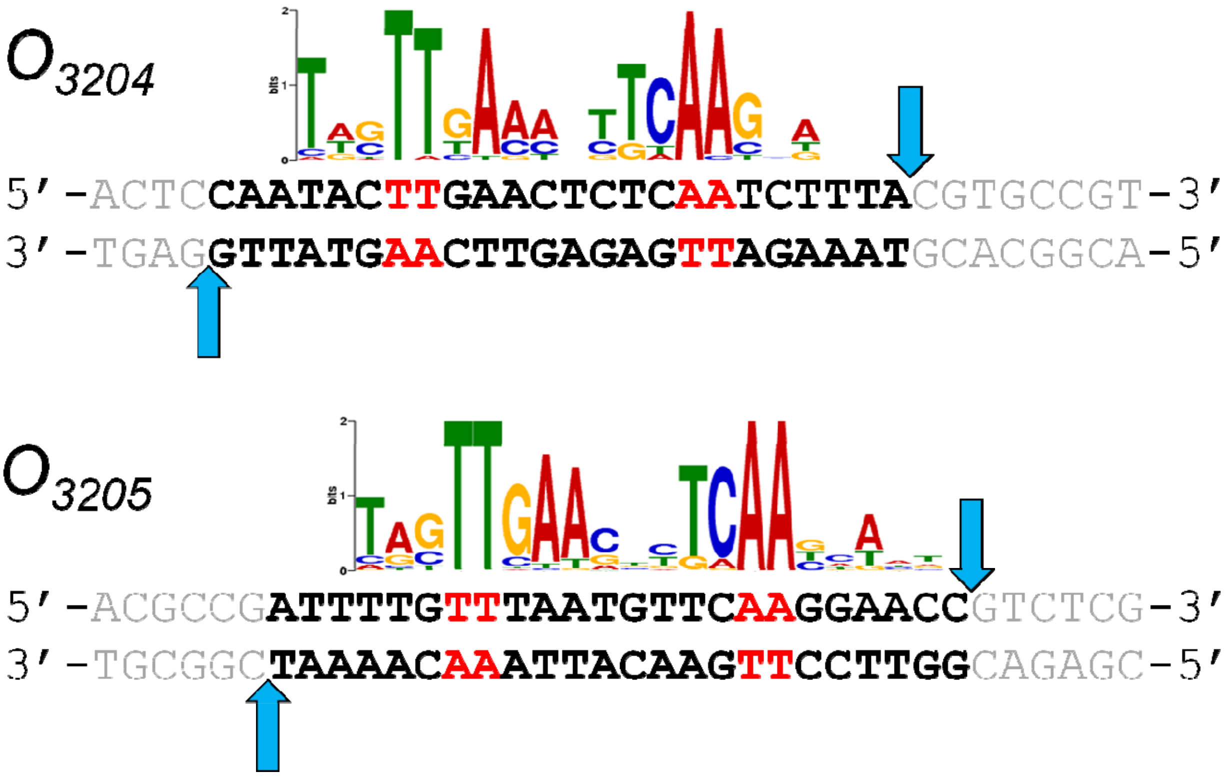

Figure S5. Footprints of O_{3204} and O_{3205} as determined by SPR compared to their corresponding MEME motifs. The full sequences shown represent the 36-mers used at the start of the footprinting procedure, and the blue arrows indicate where the boundaries were chosen as a result of these experiments. The prominent TT/AA palindromic sequence present in both operators is highlighted in red.

A

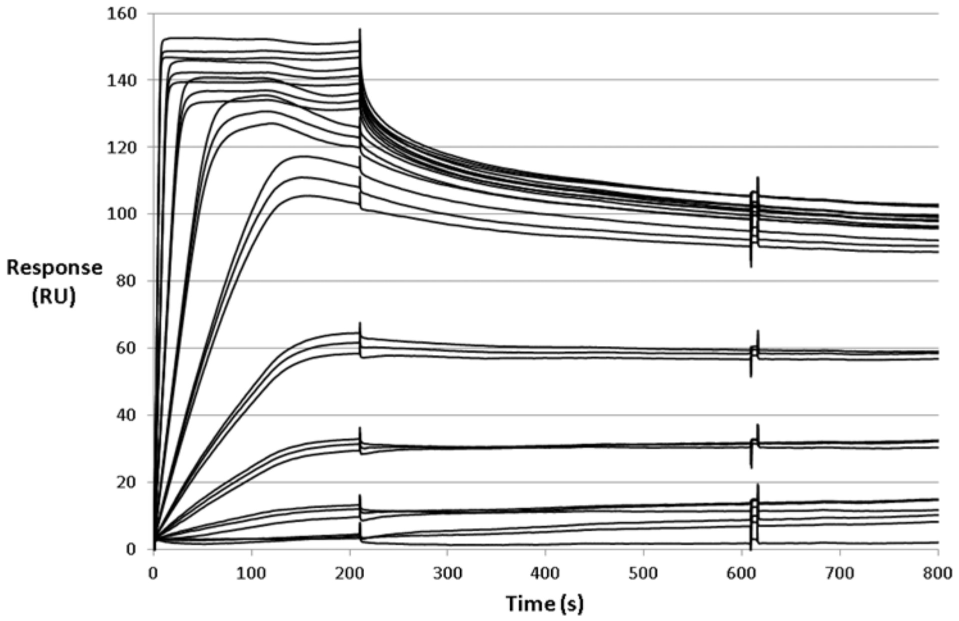

B

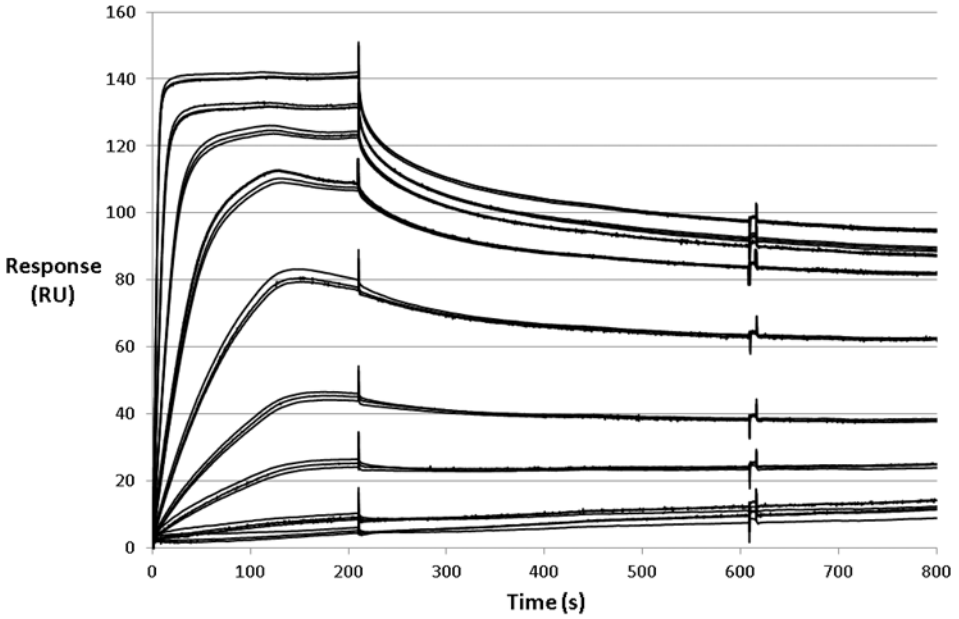

Figure S6. SPR sensorgrams showing affinity measurements for SCO3205 binding to (**A**) O_{3204} and (**B**) O_{3205}. For both sequences, the test DNA was re-captured for each cycle. A range of SCO3205 concentrations was used (0.39, 0.78, 1.56, 3.13, 6.25, 12.5, 25 and 50 nM) and all samples were run in triplicate together with buffer only controls. The protein binding and dissociation phases for all sensorgrams are shown.

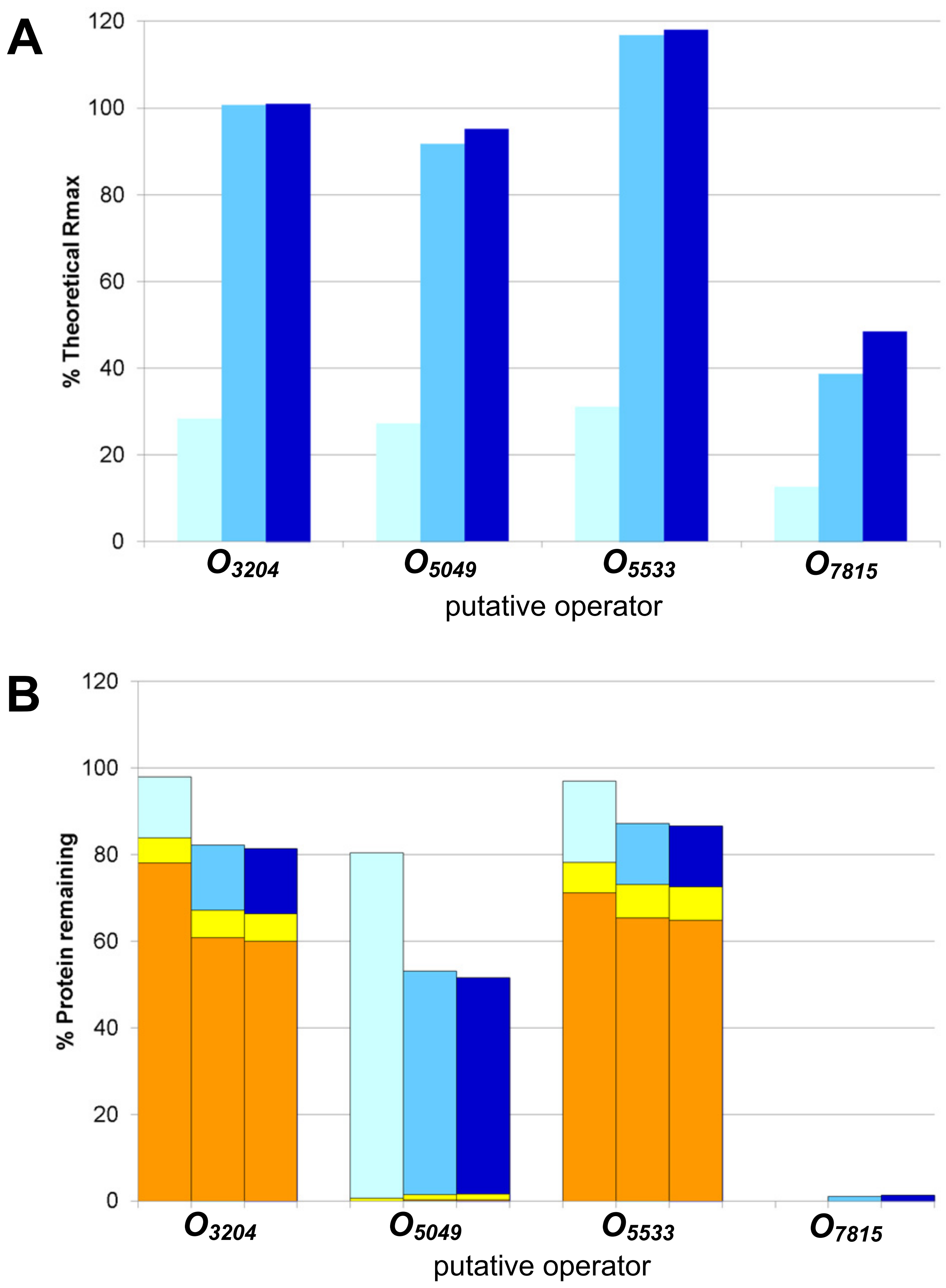

Figure S7. Binding of SCO3205 to putative regulon members. (**A**) Normalized protein binding at 10 nM (light blue), 50 nM (mid blue), and 100 nM (dark blue) is shown for each site. (**B**) The percentage of SCO3205 still bound after the dissociation phase (coloured light, mid and dark blue for 10, 50 and 100 nM SCO3205, respectively) and after each 1 M NaCl wash. In each case the first wash is coloured yellow and the second wash orange. The bars corresponding to each SCO3205 injection concentration have been overlaid.

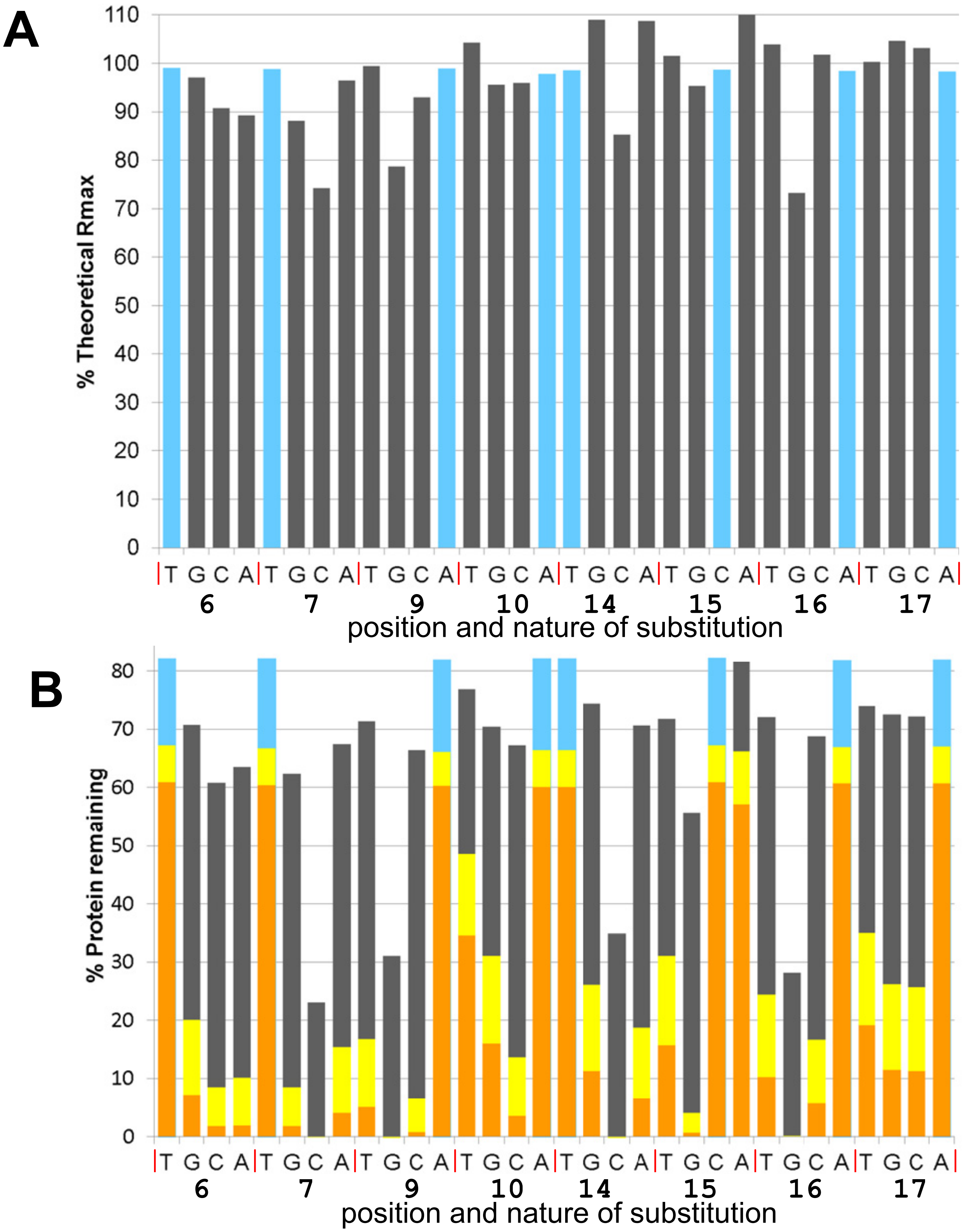

Figure S8. The effects of single substitutions within the consensus motif of O_{3204} on the binding of SCO3205. (**A**) Normalized binding of SCO3205 (at 50 nM) to each of the sequences in Supplementary Table S12. For each, the wild-type sequence is shown in blue. All the mutated sequences are shown in grey. (**B**) The percentage of SCO3205 still bound after the dissociation phase (coloured blue for the wild-type replicates, and grey for mutated sequences) and after each 1 M NaCl wash (yellow and orange, respectively). The bars for each sequence have been overlaid. The sequence numbering is relative to the 22-mer used to determine the crystal structure (see Figure 5).

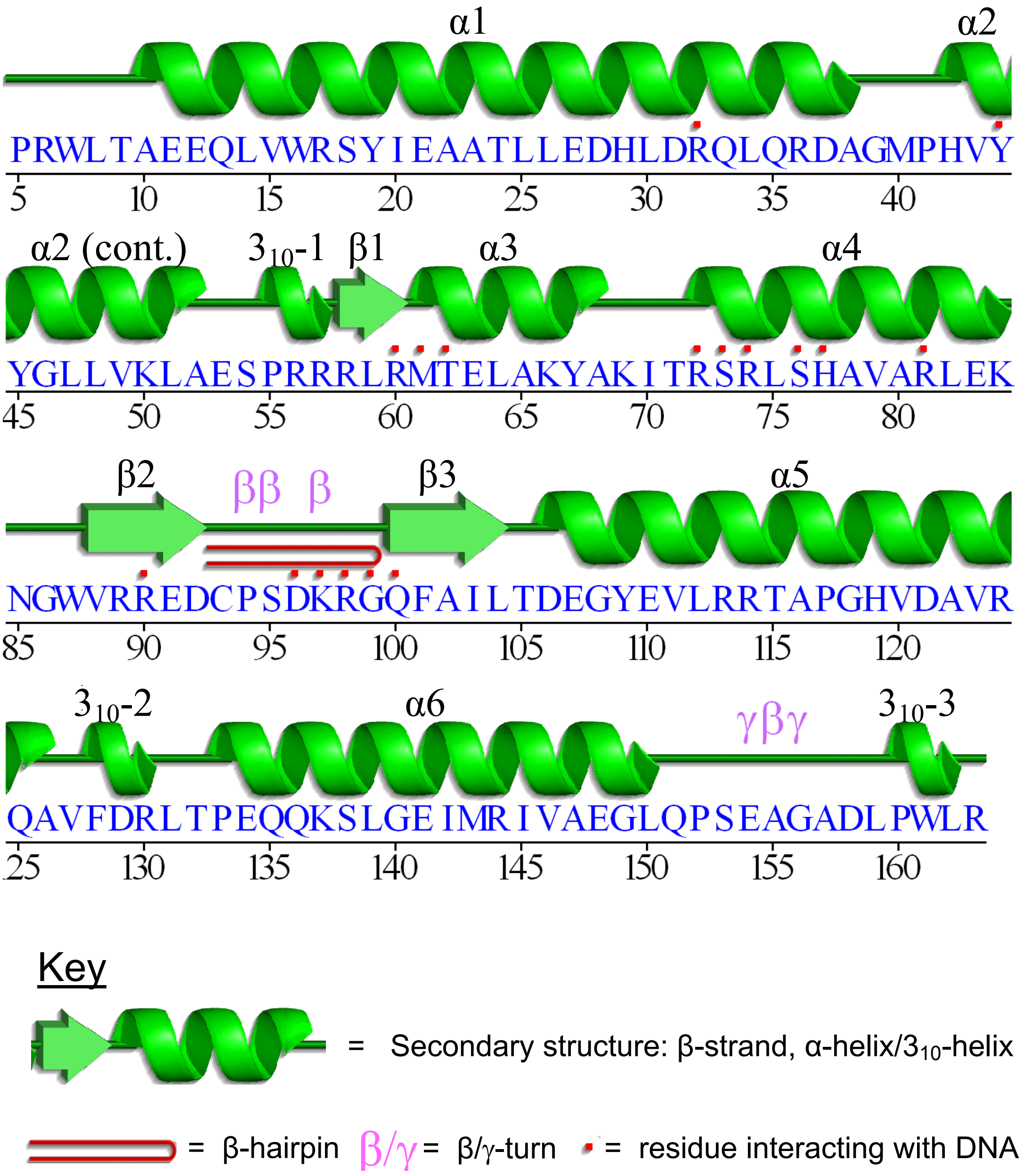

Figure S9. Secondary structure analysis of protein chain A from the SCO3205-DNA complex as output by the PDBSUM server (http://www.ebi.ac.uk/thornton-srv/databases/pdbsum/Generate.html)(54).

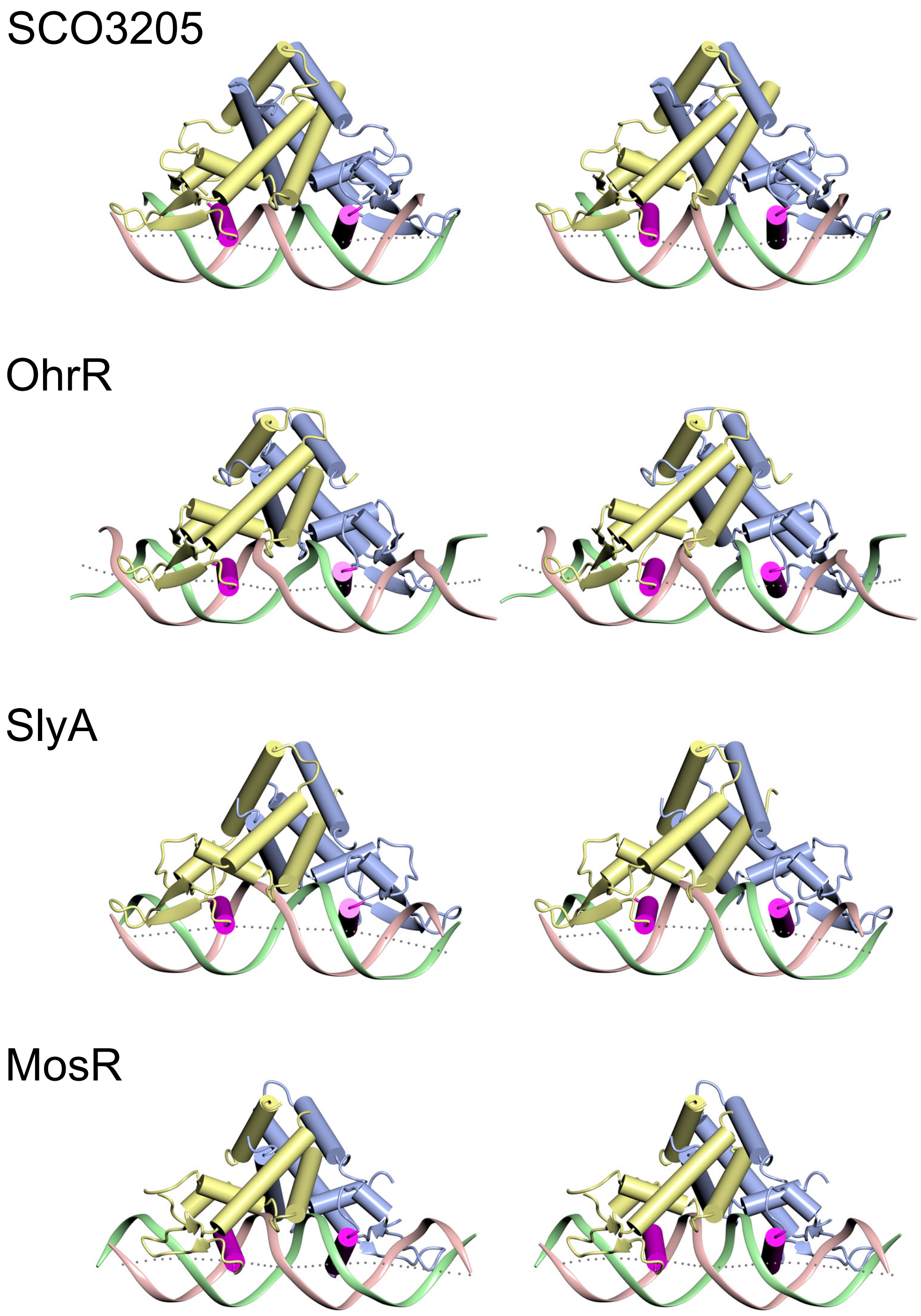

Figure S10. Cartoon stereoview representations of the SCO3205-DNA complex together with other known MFR-DNA complexes (displayed in the same relative orientation using the same colour scheme as for Figure 7). Also shown as grey dots are the helical axes for each DNA duplex as determined using CURVES+ (56).

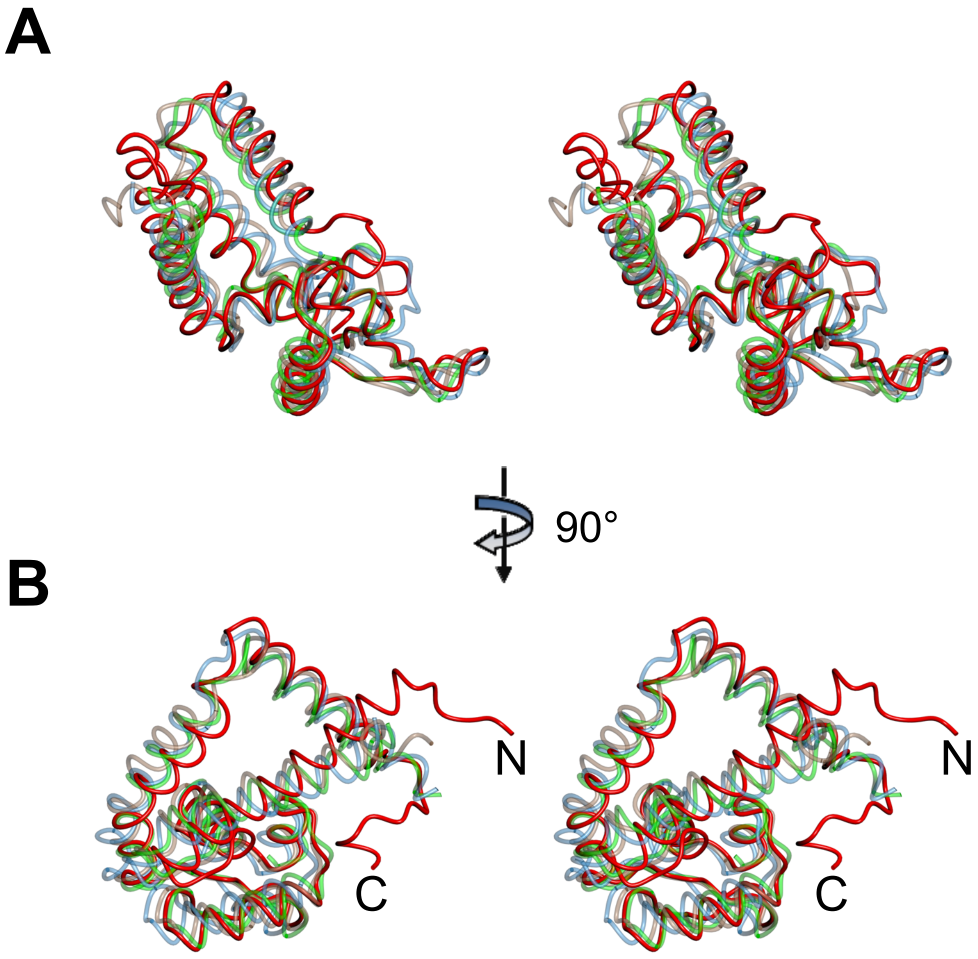

Figure S11. Stereoviews showing least-squares superpositions of the right-hand subunits taken from the MFR-DNA complexes shown in Supplementary Figure S10, (**A**) in the same orientation as in Supplementary Figure S10 and (**B**) after rotation through 90° about the vertical axis. SCO3205 is depicted in solid red, whilst the other three are shown in semi-transparent colours, where brown = OhrR, blue = SlyA and green = MosR. Note that in the SCO3205 structure both the N- and C-termini are extended relative to those of the other structures.

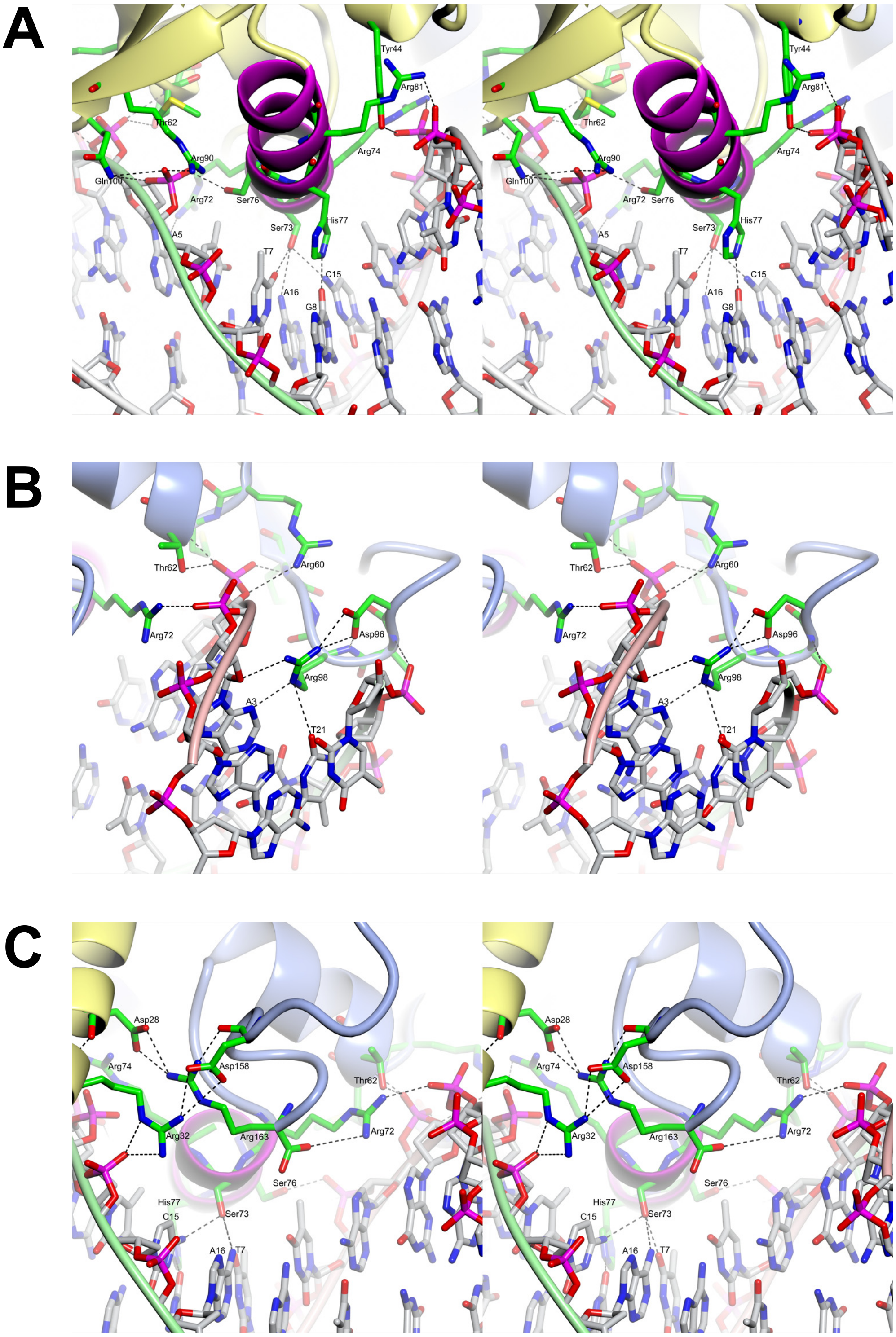

Figure S12. Stereoview close-up images focusing on key regions of the protein-DNA interface in the SCO3205-DNA complex. (**A**) Interactions between the recognition helix (α4) and the major groove. (**B**) Interactions between the wing, in particular Arg98, and the minor groove. (**C**) Indirect interactions between the C-terminal tail and the phosphate backbone: Asp158 interacts through Arg32, and the C-terminal residue, Arg163, interacts through Arg72 via its carboxyl group.

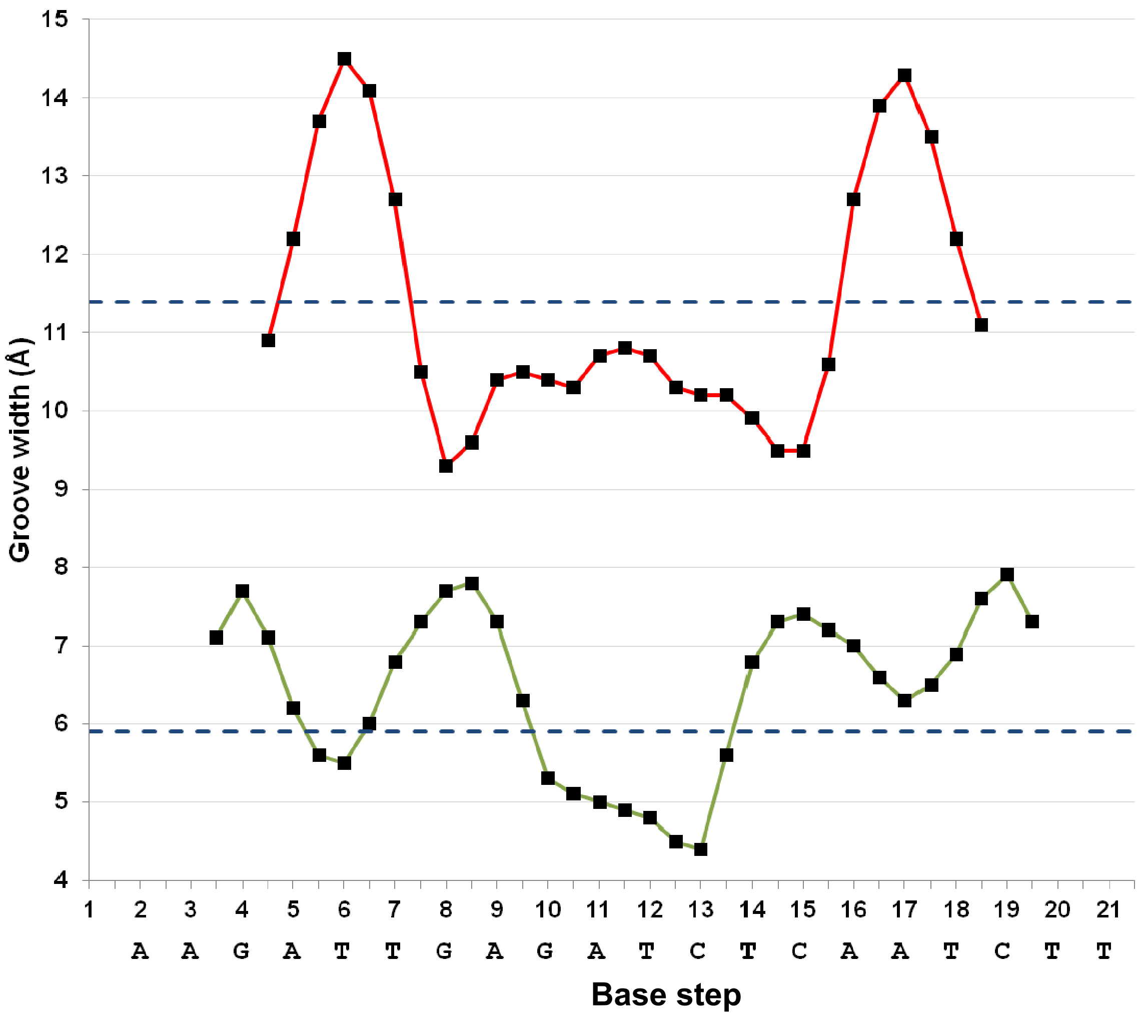

Figure S13. Widths of major (red) and minor (green) grooves for one DNA duplex in the asymmetric unit of the SCO3205-DNA complex, as calculated using the CURVES+ server (http://gbio-pbil.ibcp.fr/cgi/Curves_plus/)(56). The dashed lines show the corresponding widths in ideal B-form DNA. Very similar plots were obtained using the second DNA duplex in the ASU (data not shown).
